# Side Chain Elimination Enables Low‐Cost Fused‐Ring Acceptors and Reveals a Compact Tetrameric Structure for High‐Photocurrent Organic Solar Cells

**DOI:** 10.1002/advs.75738

**Published:** 2026-05-19

**Authors:** Jinhui Zhao, Yi Wu, Witold M. Bloch, Caroline V. I. Andersson, Leandro R. Franco, Rafael B. Ribeiro, Chuangcheng Hong, Wei Zhang, Xun Pan, Joost Kimpel, Christian Müller, Zhicai He, Bin Zhang, Ergang Wang, Mats R. Andersson

**Affiliations:** ^1^ Flinders Institute for Nanoscale Science and Technology Flinders University Adelaide Australia; ^2^ Department of Chemistry and Chemical Engineering Chalmers University of Technology Gothenburg Sweden; ^3^ School of Materials Science and Engineering Zhengzhou University Zhengzhou China; ^4^ Laboratory for Chemistry of Novel Materials University of Mons Mons Belgium; ^5^ Institute of Physics University of São Paulo Brazil; ^6^ School of Physics and Materials Science Guangzhou University Guangzhou China; ^7^ Institute of Polymer Optoelectronic Materials and Devices State Key Laboratory of Luminescent Materials and Devices South China University of Technology Guangzhou China; ^8^ Engineering Research Center for Energy Conversion and Storage Technology of Guizhou Guizhou University Guiyang China

**Keywords:** cost‐effective, fused‐ring electron acceptors, organic solar cells, single crystal, synthetic complexity index

## Abstract

Developing low‐cost, high‐performance non‐fullerene acceptors is crucial for the commercialization of organic solar cells (OSCs). Here, we introduce a β‐side‐chain‐free molecular design strategy that enables the preparation of two new Y6 derivatives, JSM5 and JSM6, with substantially reduced synthetic complexity and cost. Single‐crystal X‐ray analysis reveals an unusual tetrameric packing, originating from the removal of the β‐alkyl chains in the structure. Importantly, this packing unlocks multiple S···N intermolecular interactions between the benzothiadiazole cores, driving γ‐shaped molecular conformations to establish efficient layer‐by‐layer 3D charge transport pathways and exhibit superior intermolecular connectivity. Consequently, remarkably high short‐circuit current densities (*J*_SC_s) of up to 28 mA cm^−2^ are obtained, among the highest reported for Y‐series acceptors. PM6:JSM5 and PM6:JSM6 OSCs achieve power conversion efficiencies of 17.3% and 18.0%, respectively, comparable to the state‐of‐the‐art acceptors Y6 and BTP‐eC9, while reducing acceptor material cost by approximately threefold. Cost–performance analysis further indicates that JSMs rank among the most cost‐effective fused‐ring acceptors within the evaluated set. This work not only introduces a new packing motif enabled by β‐side chain elimination but also establishes a promising molecular design framework for high‐*J*_SC_, cost‐effective fused‐ring acceptors, paving the path toward commercially realizable acceptor materials.

## Graphical abstract

β‐side chain elimination enables cost‐effective fused‐ring acceptors, JSM5 and JSM6, with simplified synthesis. The observed compact tetrameric structures induced by S···N intermolecular interactions lead to 3D charge transport and enhance intermolecular connectivity, delivering high short‐circuit current density  up to 28 mA cm^−2^, a step closer to the theoretical upper limit, and contributing to high power conversion efficiencies of 17.3%–18.0%. Their material cost is reduced to one‐third of Y6/BTP‐eC9 while retaining high efficiency.

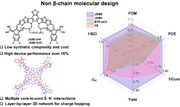

## 1 Introduction

**FIGURE 1:**
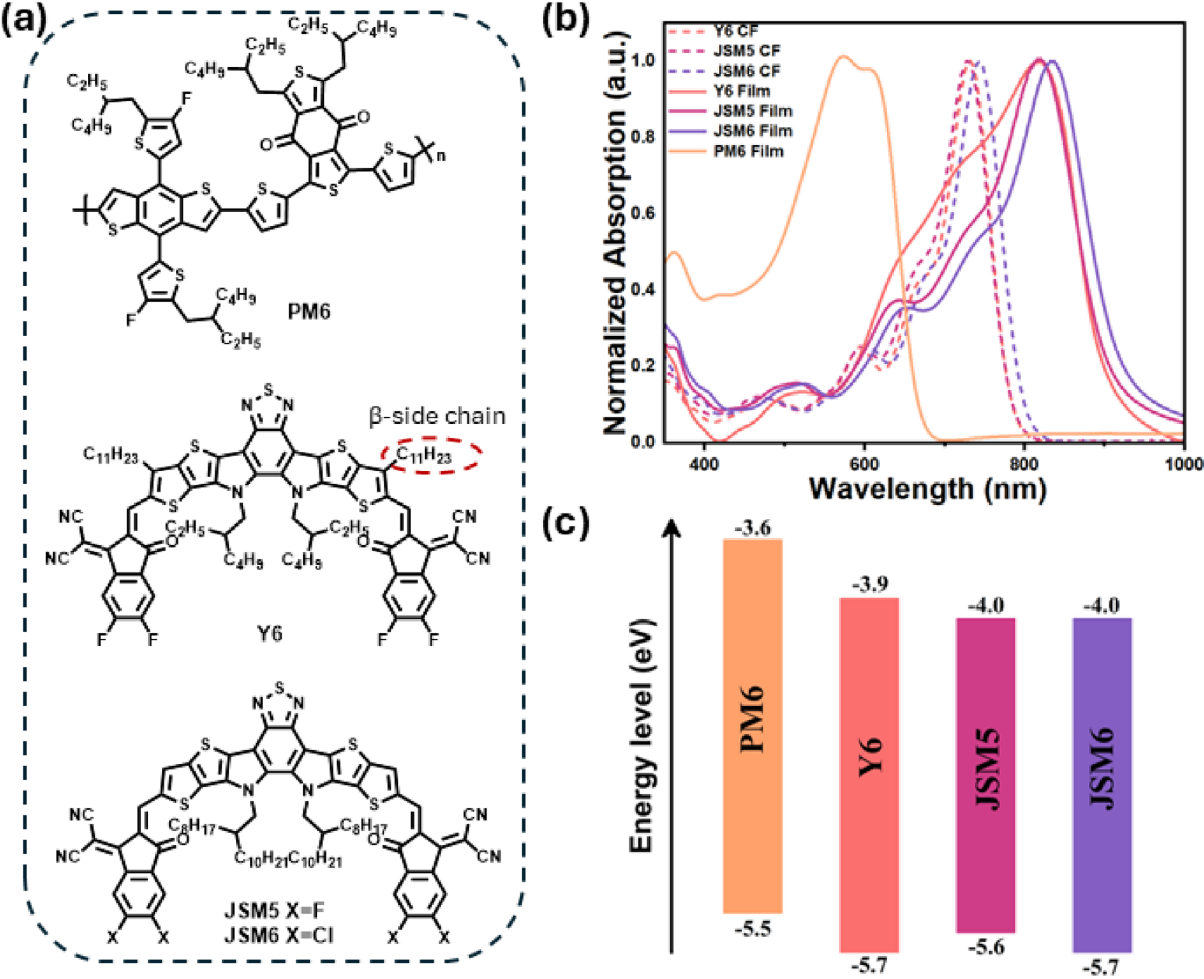
(a) Structures of polymer donor PM6, and acceptors Y6 and JSMs. (b) The normalized UV–vis spectra of the two acceptors in chloroform (CF) and the solid state. (c) Energy levels of PM6 and the acceptors.

Since the emergence of the fused‐ring electron acceptor (FREA) Y6 (Figure 1a) in 2019, advancements in the field of cutting‐edge organic solar cells (OSCs) have been dominated by developments of Y‐series acceptor derivatives [1, 2, 3, 4]. Through considerable effort in molecular design and modification to modulate energy levels, absorption spectra, and morphology, FREAs have progressively ushered organic photovoltaics into a new era with increased power conversion efficiencies (PCEs) exceeding 20% [5, 6, 7, 8]. However, the advancement in PCE has been accompanied by a growing synthetic complexity index (SCI), along with increasing synthesis costs [9, 10]. Given the importance of low‐cost acceptor materials for the commercialization of OSCs, Huang et al. pioneered the research on cost‐effective non‐fused‐ring electron acceptors (NFREA), combining conformational locks, triggered by S⋯O, O⋯H, and H⋯F interactions, to overcome insufficient molecular backbone rigidity [11, 12, 13]. Despite the incorporation of the 2‐(3‐oxo‐2,3‐dihydroinden‐1‐ylidene) (IC) end groups to extend conjugation and strengthen molecular aggregation, NFREAs tend to form large intermixed domains with the donor polymer originating from their weak intrinsic crystallinity, which leads to unfavorable charge recombination and lower fill factor (FF) [14]. Consequently, the associated device performance still lags behind that of the cutting‐edge FREAs.

The pursuit of low‐cost NFREAs has promoted the need for acceptor materials that combine low synthetic complexity and cost with high photovoltaic performance. However, for Y‐series derivatives, a major contributor to their high material cost is the incorporation of β‐alkyl side chains on the fused central core. β‐Side chain engineering, although widely used to tune solubility, crystallinity, and morphology, is always associated with early‐stage functionalization, additional synthetic steps, and complicated purification [15, 16, 17, 18, 19]. For example, Sun et al. reported L8‐BO with branched β‐side chains, boosting the PCE from 16.6% to 18.3% on average; however, this improvement required three additional reactions and seven extra purification steps, leading to a large rise in synthetic complexity and raw materials cost [20, 21]. Kong et al. further quantified this issue by showing that the cost per kilogram (*C*_kg_) of L8‐BO and BTP‐eC9 reaches 272 × 10^3^ and 212 × 10^3^ USD kg^−1^, respectively [22]. Thus, despite their excellent efficiencies, meaningful scale‐up of most cutting‐edge Y‐series acceptors remains constrained by the cost increment inherent to β‐side chain modification. To address these challenges, we sought to design fused‐ring acceptors that retain the desirable optoelectronic and morphological advantages of Y‐series molecules while fundamentally reducing synthetic complexity and cost. Our strategy is to eliminate β‐side chains on the thienothiophene units, dramatically improving yields while alleviating steric hindrance within the molecular backbone. Earlier work by Woo et al. showed that removing β‐side chains in Y6 analogues strengthens core–core and terminal–terminal (CC–TT) packing and maintains high device efficiency [23]. However, their leading candidate, YBO‐FO, still required asymmetric design and complicated purification, which limited its scalability.

Based on these insights, we designed two β‐side‐chain‐free FREAs, herein named JSM5 and JSM6, by pairing the central core with widely used IC‐2F and IC‐2Cl end groups (Figure 1a) [1, 2], which can promote strong *π*–*π* stacking interactions [24, 25, 26] and avoid the challenges of purifying isomers encountered with mixed halogen patterns [27, 28, 29, 30]. Moreover, IC‐2Cl is more synthetically available than IC‐2F, further reducing cost. Compared with β‐alkylated Y6, JSM molecules exhibit a stronger intramolecular charge transfer (ICT) character, narrower optical bandgaps (Figure 1b), and significantly enhanced molecular aggregation. Single‐crystal X‐ray analysis reveals that the elimination of β‐side chains enables multiple S···N interactions from the central benzothiadiazole units, inducing an unusual compact tetrameric packing motif. This tetrameric arrangement forms a well‐defined layer‐by‐layer crystal network that is expected to show superior intermolecular connectivity and facilitate 3D charge transport, which is supported by its high electron mobility in neat films and well‐balanced electron/hole mobilities in blend films. As a result, it likely contributes to the remarkably high short‐circuit current densities (*J*_SC_s) up to 28 mA cm^−2^, a step closer to the theoretical upper limit (∼35 mA cm^−2^ at 1.35 eV bandgap) [31, 32]. PM6:JSM5 and PM6:JSM6 devices achieve PCEs up to 17.3% and 18.0%, outperforming the β‐alkylated counterparts Y6 and BTP‐eC9. Importantly, this kind of molecular structure contributes to significantly lower material cost. The *C*_kg_ values of JSM5 and JSM6 are only 84 × 10^3^ and 77 × 10^3^ USD kg^−1^, respectively, corresponding to approximately one‐third of the cost of Y6 and BTP‐eC9. The quantified result demonstrates that JSMs are among the most cost‐effective fused‐ring acceptors reported, based on the analysis methods used. This work demonstrates the use of β‐side‐chain‐free FREAs for constructing low‐cost active layers and provides fundamental insights for designing cost‐effective acceptors.

## 2 Results and Discussion

Although the elimination of β‐side chains does not reduce the number of synthetic steps compared with Y6 and BTP‐eC9 (Schemes S1–S4), the overall yields for the synthesis of the β‐side‐chain‐free acceptors JSM5 and JSM6 are more than three times higher compared to their β‐alkylated counterparts. Therefore, the synthetic costs are significantly reduced, which will be discussed in the cost evaluation section in detail. Notably, JSM6 requires one less synthetic step than JSM5, making it a more cost‐effective acceptor [32]. Considering the absence of β‐side chains, the length of the *N*‐alkyl chains was extended to ensure good solubility in organic solvents. As a result, JSM5 and JSM6 can be readily dissolved in common solvents such as dichloromethane (DCM) and chloroform (CF), allowing for good processability during device fabrication. The molecular structures were confirmed by ^1^H‐NMR and High‐Resolution Mass Spectrometry (Figures S4–S6).

We utilized UV–vis absorption spectroscopy to investigate the optical characteristics of JSMs and Y6 in CF and of their respective thin films (Figure 1b). In solution, both β‐side‐chain‐free acceptors show strong and sharp peaks in the range of 630–800 nm, with the absorption peak of JSM6 being more red‐shifted by 16 nm. Similar effects are observed in the solid state, along with an onset above 900 nm. The corresponding optical bandgaps (*E*_g_^opt^) are 1.36 and 1.35 eV for JSM5 and JSM6, respectively (Table 1). Y6 has a similar and slightly red‐shifted absorption in CF solution compared with JSM5, while the absorption in the solid state displays an identical peak shift but a wider shoulder peak compared with JSM5. This can be attributed to the extra intermolecular interactions of Y6 [2]. The low‐lying absorption onset of Y6 films corresponds to an optical bandgap of 1.37 eV. JSMs and Y6 possess complementary absorption spectra with PM6. Additionally, the intensity ratio of 0–1 to 0‐0 peak in the solid state is a measure of ICT effect and molecular aggregation [33]. The calculated intensity ratio of JSM6 is higher than that of JSM5, indicating enhanced molecular aggregation and ICT in the JSM6 film. Y6 exhibits a much lower intensity ratio than those of JSMs. We attribute the weakened ICT to electron donation from the β‐side chains. The results collectively illustrate that the absence of β‐side chains can further promote the aggregation of Y‐series acceptors, and the effect of chlorine atoms on facilitating molecular aggregation also applies to β‐side‐chain‐free FREAs in both solution and thin films [1, 34]. In addition, Time‐Dependent Density Functional Theory (TD‐DFT) calculations were performed to estimate the UV–vis absorption (Table S2). We observe that JSM5 and Y6, both with fluorine substitutions, exhibit comparable peak shifts, while JSM6 exhibits a more red‐shifted absorption (Figure S7), consistent with a narrower fundamental gap of 1.80 eV and indicative of enhanced ICT, which aligns with the UV–vis result. The calculated bandgaps of JSMs and Y6 follow the same trend as indicated by UV–vis spectra.

**TABLE 1:** Summary of optical properties for JSMs and Y6.

Acceptor	*λ*_max_ solution (nm)	*λ*_max_ film^a^ (nm)	*λ*_onset_ (nm)^b^	*E*_g_^opt^ (eV)	0–1 peak (nm)	Ratio^c^
Y6	732	818	901	1.37	716	1.39
JSM5	728	818	910	1.36	730	1.78
JSM6	744	836	921	1.35	740	1.89

^a^ 0‐0 peak of absorption in film.

^b^ The onset of absorption in a solid film.

^c^ The ratio of 0–1 peak intensity to 0‐0 peak.

Cyclic voltammetry (CV) was used to evaluate the energy levels of the molecules (Figure S8), and the HOMO/LUMO levels were estimated to be −5.6 eV/−4.0 eV, −5.7 eV/−4.0 eV, and −5.7 eV/−3.9 eV for JSM5, JSM6, and Y6 (Figure 1c), respectively. Overall, the β‐side‐chain‐free JSMs exhibit slightly deeper energy levels than Y6, consistent with the reduced electron‐donating contribution from β‐alkyl substituents. Importantly, the trend is consistent with DFT calculations (Table S2), which predict stabilization of the LUMO for JSMs relative to Y6 and a small additional stabilization upon chlorination in JSM6. Minor deviations in the absolute values between CV and DFT are expected, given the different reference conditions (electrochemical oxidation/reduction in the solid state vs computed gas/continuum models), and do not affect the qualitative conclusions regarding energy‐level alignment.

To study the molecular aggregation behavior inferred from UV–vis spectra, grazing‐incidence wide‐angle X‐ray scattering (GIWAXS) was conducted on neat JSM5 and JSM6 films to assess molecular packing (Figure S9). The face‐on orientation in the out‐of‐plane (OOP) direction dominates the molecular packing for both films. The *π*–*π* stacking peaks for JSM5 and JSM6 are located at 1.82 Å^−1^ (*d* = 3.47 Å) and 1.81 Å^−1^ (*d* = 3.45 Å), with corresponding coherence lengths (CCLs) of 15.7 and 17.6 Å, respectively. Y6 also exhibits a predominant face‐on orientation (Figure S9), and a larger *π*–*π* stacking distance of 3.57 Å [2]. These results reveal that the β‐side‐chain‐free molecular backbone contributes to compact *π*–*π* stacking, and the chlorinated JSM6 has the most pronounced molecular packing in the vertical direction. In the in‐plane (IP) direction, lamellar stacking signals of (100) and (200) can be observed for neat films of JSM5 and JSM6. The intense (100) and (200) peaks in JSM6 film are located at *q*_xy_ = 0.36 and 0.25 Å^−1^, respectively, with corresponding *d*‐space of 17.0 and 25.0 Å. In case of JSM5, the (100) peak is located at *q*_xy_ = 0.36 Å^−1^, and a weak (200) peak at *q*_xy_ = 0.25 Å^−1^ can be found, with corresponding *d*‐space of 17.5 and 25.2 Å, showing comparable crystalline ability with JSM6 in the IP direction. The result suggests that the absence of β‐side chains can lead to more compact molecular packing in films due to efficient *π*–*π* interactions.

To deepen the understanding of how the absence of β‐side chains affects molecular packing, we grew single crystals of JSM5 and JSM6 through the ternary solution diffusion method with CF, DCM, and methanol. Single‐crystal X‐ray diffraction was performed to analyze the molecular geometry and intermolecular interactions. Y6 crystallizes in the monoclinic space group P2_1_/*c* [24], JSM5 crystallizes in the orthorhombic space group C222_1_, and JSM6 crystallizes in a triclinic crystal system with a P‐1 space group (Table S3), reflecting that JSM5 possesses the highest symmetry of unit cells among all FREAs to date [24, 25, 26, 27, 28]. From the unit cell of crystals, we can observe several recurrent motifs. Both unit cells of JSM5 and Y6 exhibit two types of molecular conformations, and four types of conformations are observed for the JSM6 unit cell (Figure S10). The molecular backbones of JSMs show identical C‐shape geometry as for Y6, and their intramolecular S⋯O distances are similar (Table S4). Notably, the torsion angles of JSM6 *π*‐cores are much larger than those of Y6 and JSM5, reflecting a more twisted molecular backbone (Figure S11). This can be attributed to the stronger end‐group interaction induced by the chlorine atoms [34].

**FIGURE 2:**
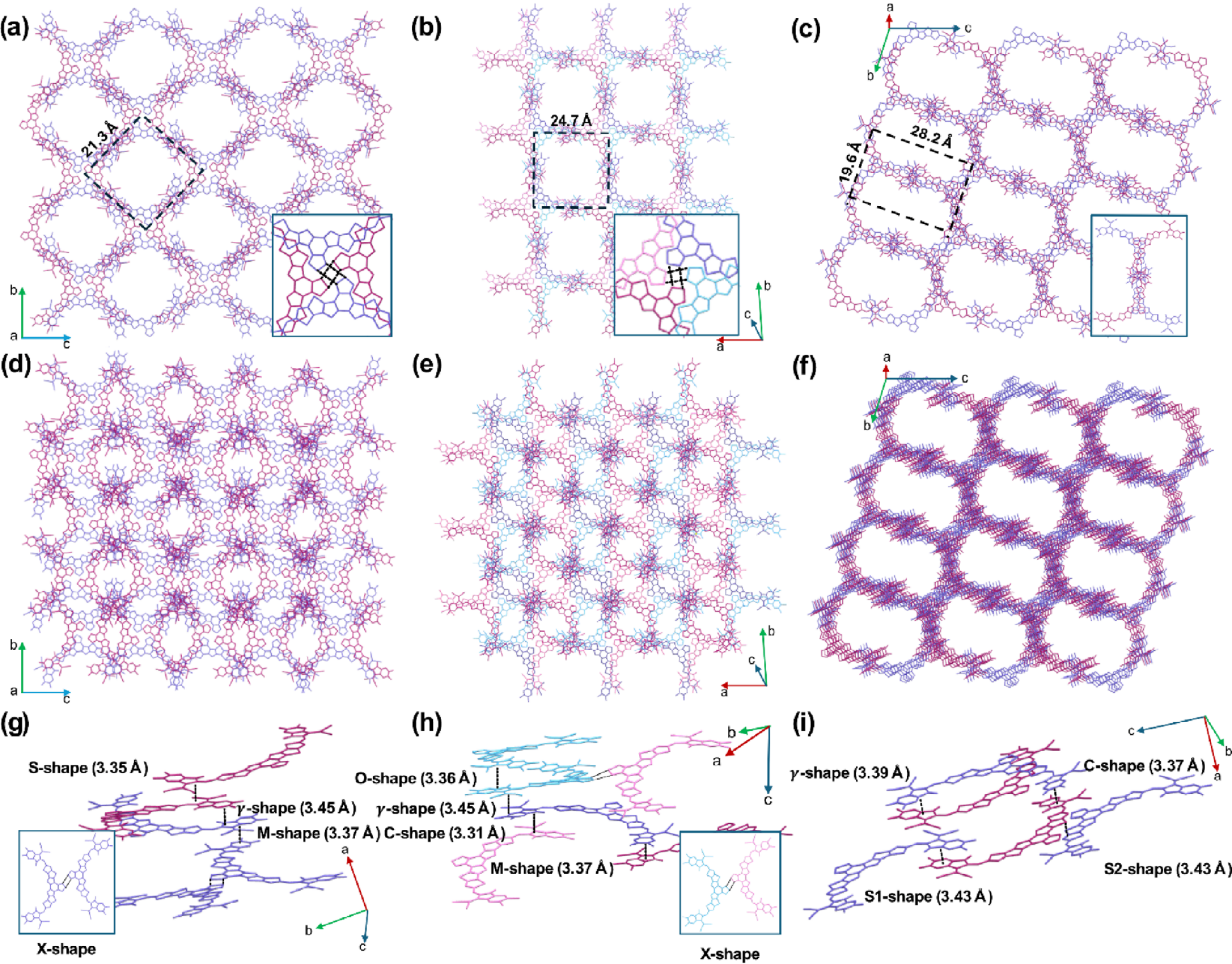
Mono‐layer networks of (a) JSM5, (b) JSM6, and (c) Y6; (a, b) display S⋯N intermolecular interactions within the tetrameric structures. Multilayer networks of (d) JSM5, (e) JSM6, and (f) Y6. Units containing dimers induced by *π*–*π* stacking and S⋯N interactions within (g) JSM5, (h) JSM6, and (i) Y6. Alkyl chains were removed to increase the visibility of the packing. Note that Y6 has many polymorphs due to different solvent systems for crystal growth. Here, the representative Y6 crystal we chose was also prepared out of CF [24, 35]. The packings observed in the single crystals do not fully represent the packing in the active layer of the devices.

Dimers induced by *π*–*π* stacking interactions account for the majority in all units (Figure S12), M‐shaped TT1 and S‐shaped TT2 in JSM5, M‐shaped TT1, C‐shaped TT2, and O‐shaped TT3 in JSM6, and C‐shaped TT1, S1‐shaped TT2, and S2‐shaped CT‐CT in Y6 [24]. Notably, the γ‐shaped CC‐TT dimer, characterized by not only a *π*–*π* stacking interaction between end groups but also a face‐to‐face *π*‐core interaction between molecular backbones, can also be found for JSMs. This kind of packing mode is commonly observed in single crystals of Y‐series derivatives and has been demonstrated to facilitate efficient exciton dissociation and charge transfer due to efficient molecular overlap, leading to improved charge generation and device performance [35, 36, 37]. Within these stacked motifs, the average *π*–*π* stacking distance is 3.39, 3.37, and 3.40 Å for JSM5, JSM6, and Y6 (Figure 2g–i), respectively, in agreement with the trend from GIWAXS of neat films.

Apart from *π*–*π* stacking induced dimers, we also observe coplanar dimers for both JSMs and Y6 induced by non‐covalent interactions. Interestingly, we identified an X‐shaped dimeric conformation for both JSMs (Figure 2g,h), and the presence of β‐side chains plays a critical role in determining this dimeric conformation, as the removal of large steric hindrance associated with β‐side chains in JSMs allows the participation of dual parallel intermolecular S⋯N interactions that align a coplanar X‐shaped dimeric conformation. Importantly, we observed that the extended *π*–*π* network of JSMs is constructed by repeating a tetrameric unit (Figure 2a,b). Despite the similarities in the *π*‐conjugated molecular structures of many FREAs, such a tetrameric unit has not been previously observed in those single crystals [24, 25, 26]. The underlying reason could be the significantly reduced steric hindrance associated with side chains. This tetrameric structure also appears in the crystal packing of JSM6. However, the unit cell of JSM5 appears to be half of the tetrameric structure. We attribute the formation of the tetrameric structure in JSM5 to the identified multiple S⋯N intermolecular interactions between benzothiadiazoles from different molecules. The main contributing factor leading to S⋯N interactions is the electron donation from a nitrogen lone pair of one unit into the S‐N antibonding orbital on another unit ($n_{N}\to \sigma_{S-N}^{*}$). This is supported by the nearly 180° angle between these atoms as well as the elongation of S─N bonds involved in the interaction (Figure S13a–f). JSM5 crystals feature a better orbital overlap, with an N⋯S‐N angle of 172.7°, as compared to JSM6, with an N⋯S‐N angle of 170.2° due to its twisted backbone. This can be ascribed to the stronger *π*–*π* interaction from IC‐2Cl end groups induced by the enhanced *π*‐electron delocalization from chlorine substituents [34]. The orbital overlap is reflected in the S─N bond length increase: JSM5 observes an average elongation of 0.0465 Å for the S─N bonds involved in intermolecular interaction, whereas JSM6 only experiences an average increase of 0.0013 Å for the same bond, indicating that JSM5 showcases stronger S⋯N intermolecular interactions. Therefore, we believe that there is a trade‐off between these two intermolecular interactions, and the dual parallel S⋯N intermolecular interactions between JSM5 molecules lead to the observed tetrameric structure. In addition, we measured the stacking distances of end groups and *π*‐cores. The average *π*–*π* stacking distances of IC‐2F and IC‐2Cl are 3.426 and 3.410 Å, respectively, demonstrating a stronger *π*–*π* stacking between IC‐2Cls in JSM6, which is consistent with GIWAXS results. The distance between *π*‐cores is quantified by measuring the S‐N distance (Figure S13). The S‐N distances within JSM5 crystals are identical (3.684 Å), whereas the average distances of those of JSM6 are 3.889 Å, reflecting a flatter tetrameric structure of JSM5.

The single‐crystal structures of JSM5 and JSM6 exhibit highly ordered *π*–*π*‐stacked molecular domains (Figure 2a–c). The interstitial regions between these ordered domains form well‐defined uniaxial porous channels that are occupied by disordered alkyl chains. In the β‐side‐chain‐free systems, the channels adopt an approximately circular geometry, inscribed within squares with side lengths of approximately 21.3 and 24.7 Å for JSM5 and JSM6, respectively. By contrast, the channels in Y6 display a double‐lens‐like geometry, inscribed within a rectangle measuring approximately 19.6 by 28.2 Å. These channels have a diameter of 21.3 and 24.7 Å for JSM5 and JSM6, respectively, showing comparable sizes to that of Y6 (19.6 Å × 28.2 Å). Notably, the identified tetrameric units of both JSM5 and JSM6 efficiently leverages γ‐shaped dimeric conformations, which we believe can enhance exciton transport and charge transfer [38]. To investigate the conformational impact on charge transport, we performed DFT calculations to evaluate the electronic couplings between molecules in representative dimeric configurations for both electron and hole transfer within the crystal lattice. Our analysis reveals a small number of recurring dimer motifs (Figure S12), characterized by offset contacts and minimal orbital interaction. It can be found that the *π*‐stacked dimeric conformations show predominantly high charge transfer couplings, whereas non‐*π*‐stacked dimers exhibit negligible coupling values (Table S6). All *π*‐stacked dimers of JSM5 display the lowest coupling values on average, whereas those of Y6 have the highest values. As charge transfer is generally described in the semiclassical regime using Marcus‐type equations [39, 40, 41], one would, as a first approximation, based on hopping between a pair of close molecules, expect similar charge‐transfer rates for JSM6 and Y6 (Tables S7 and S8). However, our space‐charge‐limited current (SCLC) measurements on neat films reveal that Y6 exhibits the lowest electron mobility (*µ*_e_) among the three materials. The experimental *µ*_e_ values are 4.81 × 10^−4^ and 5.95 × 10^−4^ cm^2^ V^−1^ s^−1^ for Y6 and JSM5, respectively, while JSM6 shows the highest *µ*_e_ of 7.51 × 10^−4^ cm^2^ V^−1^ s^−1^ (Table S9). Although dimers with extremely low couplings can hinder the charge hopping and affect the mobility, we believe that the measured differences in *µ*_e_ are primarily due to the lattice connectivity. As shown by the monolayers, each dimer is a building block of the lattice (Figure 2a–c), where electrons can hop along the continuous pathways provided within the network. Interestingly, as we deepen the axis of the crystalline network (axis a of the JSM5 crystal and axis c of the JSM6 crystal in Figure 2d–g), the voids of JSMs are layered by tetrameric structures, exhibiting a more efficient layer‐by‐layer crystal arrangement compared to the ordered Y6 network. Importantly, JSM6 shows systematically the lowest internal reorganization energies (λ) for both electrons (0.114 eV) and holes (0.172 eV) (Table S2). Since reorganization energy governs the structural relaxation penalty associated with charge hopping [39], these reductions imply more flexible charge transport pathways in JSM6.

These differences in mobility can be understood via a graph‐based analysis of the intermolecular contact network [42]. By treating each molecule as a node and setting a distance cutoff to define the edges between them, we can propose a simplified percolation model to compare different crystal structures. As a result, we obtained 28, 10, and 31 components for the 2 × 2 × 2 supercells of JSM5, JSM6, and Y6 (Table S10), respectively. Each component corresponds to a subnet of molecules that can reach one another via at least one linked path, so fewer components indicate better connectivity between the molecules. This metric corresponds to the degree of network fragmentation (ϕ) and further emphasizes the greater connectivity of the β‐side‐chain‐free crystals. A higher ϕ indicates a larger number of small subnets, which supports the idea that charges can only hop efficiently between a limited fraction of molecules, as the transfer rates are significantly higher between dimers with close contacts (Figure S12 and Table S6). On the other hand, despite the lower rates, charge hopping was experimentally observed over significantly larger distances within organic semiconductor materials [43, 44], up to almost 24 Å [45]. Therefore, we varied the cutoff distance from 3 to 20 Å and compared the average number of connections per molecule, the so‐called average degree (⟨k⟩), across crystals. On average, the JSMs have more edges at 3 Å, consistent with the significant differences in network fragmentation (Figure S14). From 4 to 8 Å, ⟨k⟩ is higher for Y6, and from then on, the average degree for β‐side‐chain‐free crystals dominates until reaching the upper limit of charge hopping. Therefore, when we combine the lower ϕ at very close distances with the overall higher ⟨k⟩, we can expect a higher electron mobility in the JSM crystals, particularly in JSM6, which shows significantly higher electron transfer integrals for all dimers compared to JSM5 (Table S5).

**FIGURE 3:**
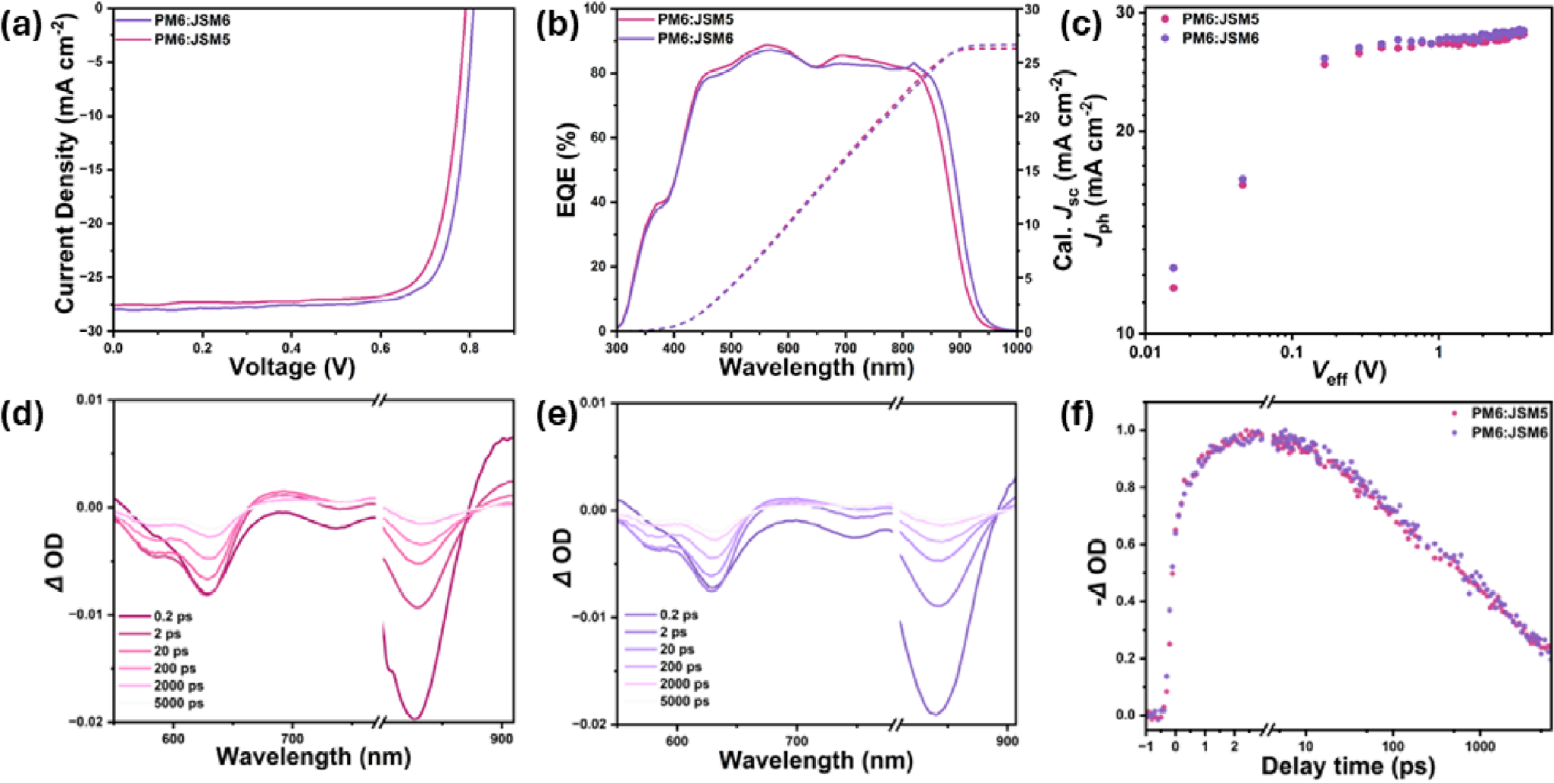
(a) *J*–*V* curves of optimal devices. (b) EQE curves of devices and integration. (c) *J*_ph_‐*V*_eff_ curves of optimal devices. Femto‐second transient absorption spectra of (d) PM6:JSM5 and (e) PM6:JSM6 devices at the indicated delay times. (f) Hole transfer kinetics of corresponding films probed at 580 nm.

We selected PM6 (Figure 1a) as the donor material to prepare blend solutions with the JSM acceptors (D: A = 1:1.2 w/w) in CF. A conventional OSC device structure of ITO/PEDOT:PSS/Active layer/PDINN/Ag was used, and the specific fabrication process is outlined in the supporting information. To achieve optimal performance, we optimized the device preparation conditions by varying the additives and the thermal annealing (TA) conditions (Tables S11–S14). After TA at 100°C for 5 min, the JSM6‐based binary device delivered a superior PCE of 18.0%, characterized by a *V*_OC_ of 0.809 V, a *J*_SC_ of 28.0 mA cm^−2^, and an FF of 0.797. A PCE of 17.3% was achieved by the PM6:JSM5 binary device with the same TA process, with a *V*_OC_ of 0.794 V, a *J*_SC_ of 27.6 mA cm^−2^ and an FF of 0.789 (Figure 3a). On average, JSM5 exhibits a comparable PCE to Y6, and JSM6 exhibits higher device performance as compared to the β‐alkylated counterpart, BTP‐eC9, under the same conditions (Table 2). JSM‐based devices exhibit outstanding *J*_SC_s, which could result from better 3D charge transport channels. We assume the obtained single‐crystal structures described above represent the most prevalent polymorphs in the studied devices. The slightly higher *V*_OC_ in the PM6:JSM6 device as compared to PM6:JSM5 could originate from the lower energy disorder of JSM6 [1]. Obviously, the lower *V*_OC_ values of JSMs‐based devices relative to Y6 are consistent with their lower LUMO levels. Similarly to the UV–vis absorption of binary films shown in Figure S15, the external quantum efficiency (EQE) spectra of two devices (Figure 3b) exhibit a broad and strong response above 80% across the range from 300 to 900 nm. The calculated *J*_SC_s are 26.4 and 26.6 mA cm^−2^ for JSM5 and JSM6 binary devices, respectively, which align well with the current density values from devices within 5% deviation.

**TABLE 2:** Optimal photovoltaic performance of PM6: JSMs devices (1:1.2, w/w).

Active layer	*V*_OC_ [V]	*J*_SC_ [mA cm^−2^]	*J*_SC_^EQE^ ^a^ [mA cm^−2^]	FF [%]	PCE ^b^ [%]
PM6:JSM5	0.794	27.6	26.3	78.9	17.3 (16.8 ± 0.2)
PM6:JSM6	0.809	28.0	26.6	79.7	18.0 (17.8 ± 0.2)
PM6:Y6	0.833	26.5	25.6	77.9	17.2 (16.9 ± 0.2)
PM6:BTP‐eC9	0.841	26.2	25.9	81.1	17.9 (17.4 ± 0.2) [1]

^a^ Integrated *J*_SC_ from the EQE curves.

^b^ The optimal PCE value with 1,3,5‐trichlorobenzene as an additive, plus the average PCE values with standard deviation from 10 devices in parentheses (Figure S15).

Stability is a multifaceted issue in organic photovoltaics, governed by morphological and interfacial stability as well as intrinsic photodegradation pathways, and is further influenced by oxygen and moisture exposure. A specific concern for acceptors lacking β‐position alkyl chains is that the exposed thiophene carbon may become a reactive site that enables cis–trans isomerization and subsequent photoinduced cyclisation under illumination, as discussed for certain ITIC‐type systems [46]. In the present Y‐series framework, however, such reactions would require disruption of strong intramolecular interactions at the terminal region and must proceed in the solid state, where conformational changes are energetically more constrained. Moreover, the enhanced molecular packing observed for JSMs is expected to further suppress end‐group rotation and isomerization. To experimentally verify that β‐side‐chain elimination does not compromise photochemical stability, we performed a photodegradation study on neat films in ambient atmosphere under simulated solar illumination (1 Sun, AM 1.5G, Figure S16). The UV–vis spectra of JSM films show only a slight initial redshift during the first ∼10 h, consistent with increased aggregation, and no emergence of new absorption bands or blue‐shifted features up to 160 h. These results indicate that the photostability of JSMs is comparable to Y6 and better than BTP‐eC9 under these conditions, supporting the viability of β‐side‐chain‐free acceptors for scalable OSC material development.

We carried out SCLC measurements to evaluate the hole mobility (*µ*_h_) and electron mobility (*µ*_e_) in blend films (Table S9). The *µ*_h_/*µ*_e_ was determined to be 9.8 × 10^−4^/9.2 × 10^−4^, 6.1 × 10^−4^/6.8 × 10^−4^, and 4.0 × 10^−4^/2.2 × 10^−4^ cm^2^ V^−1^ s^−1^ for JSM6, JSM5, and Y6 blend films, respectively. The ratio of *µ*_h_ to *µ*_e_ for JSM6 blend film is 1.1, which is comparable to 0.9 for JSM5 blend film, showing more balanced mobility than Y6 (1.8). This finding explains the high *J*_SC_ and FF for the optimized devices from JSMs.

To investigate charge generation and exciton dissociation, we carried out dependence of photocurrent (*J*_ph_) on effective voltage (*V*_eff_) experiments. Both devices exhibit high *J*_ph_ approaching 28 mA cm^−2^ (Figure 3c). The possibility of exciton dissociation and charge collection is reflected by *P*_d_ and *P*_c_, respectively. *P*_d_ is determined by the ratio of *J*_ph_ and *J*_sat_ under short circuit conditions, where *J*_sat_ is the saturated photocurrent, while *P*_c_ is calculated through the same equation under maximum power output conditions. The *P*_d_/*P*_c_ values are 97.6%/90.4% and 98.2%/90.5% for JSM5 and JSM6 devices, respectively, indicating efficient charge dynamics in both devices.

We performed femto‐second transient absorption (fs‐TAS) to gain a deeper understanding of the charge generation mechanism. An 800 nm pump beam was applied to excite the blend films. The observed ground state bleaching (GSB) peaks at 600 nm for the donor and at around 830 nm for the acceptors, mirroring the absorption peaks in Figure S15. The strong GSB signal in the acceptor region implies the excitation of excitons, and the observed excited state absorption peaks, ranging from 670 and 750 nm after GSB peaks of JSMs, indicate efficient exciton separation at the intermolecular interface. It is observed that the GSB signals decay rapidly at early timescales, followed by a new GSB characteristic peak emerging at 580 nm. This phenomenon suggests hole‐transfer from the acceptors to the donor region (Figure 3f). The corresponding kinetics on the early timescale were analyzed through the biexponential fitting of the GSB signal at 580 nm (Table S15). The τ_1_ values of both blend films are smaller than the instrument response time, suggesting that ultrafast exciton dissociation happened at the D:A interface. The τ_2_ value, representing the lifetime of exciton migration to D:A interface, were found to be 0.8 and 0.7 ps for JSM5 and JSM6 binary films, respectively, suggesting a more efficient exciton dissociation in JSM6‐based blend films.

**FIGURE 4:**
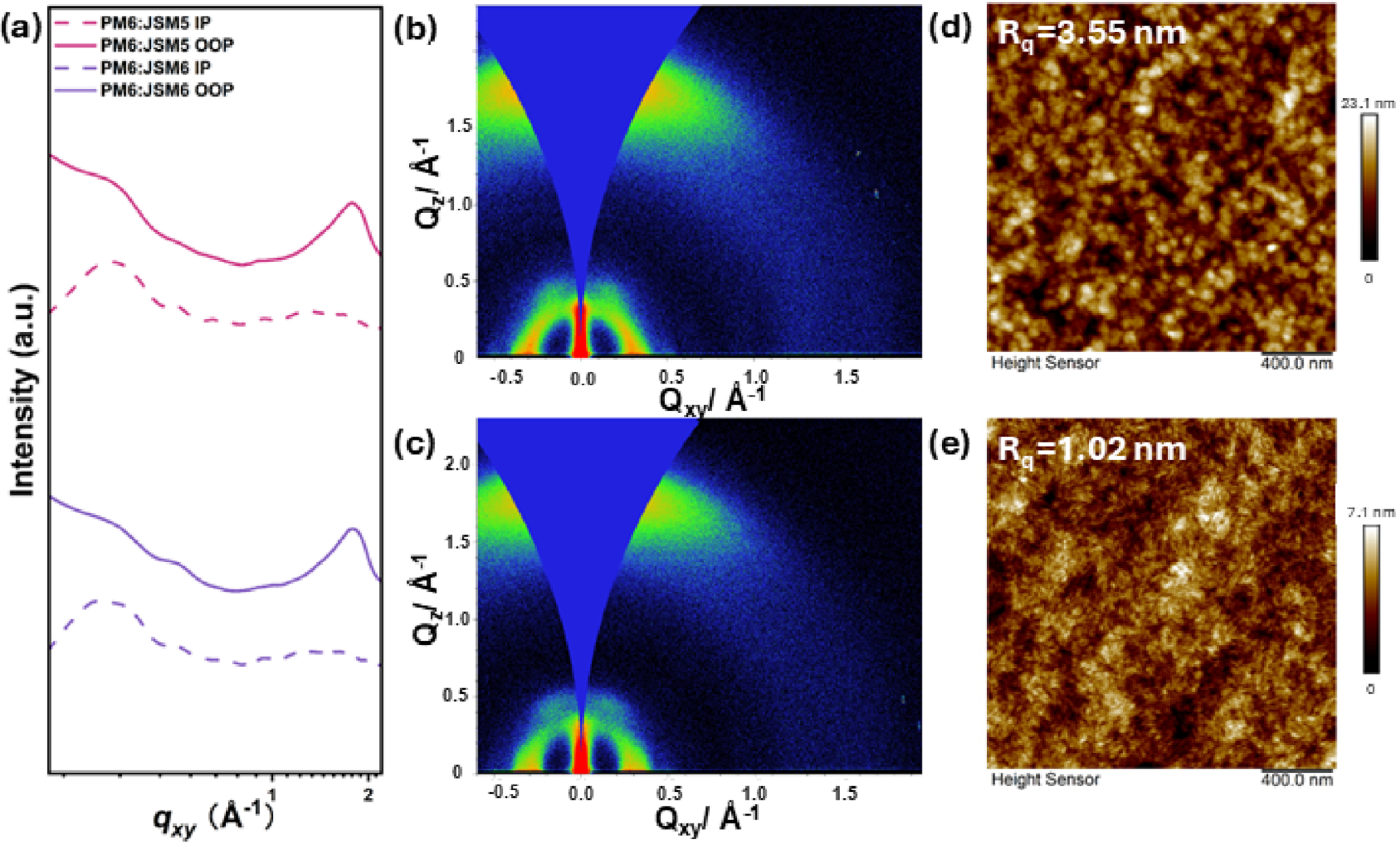
(a) 1D‐GIWAXS plot of both films. 2D‐GIWAXS patterns of (b) PM6:JSM5 blend film and (c) PM6:JSM6 blend film. AFM image of (d) PM6:JSM5 blend film and (e) PM6:JSM6 blend film with corresponding root‐mean‐square (R_q_) values.

GIWAXS measurements were conducted to gain insight into the molecular aggregation characteristics within blend films (Figure 4a–c; Table S16). In the IP direction, a lamellar packing (100) peak can be observed at *q*_xy_ = 0.30 and 0.37 Å^−1^ for JSM5 and JSM6 blend films, with corresponding *d* spaces of 20.94 and 20.37 Å, respectively. In the OOP direction, a distinct and intense signal can be observed for both blend films, featuring a predominant face‐on orientation to the substrate. The *π*–*π* stacking diffraction peaks (010) for JSM5 and JSM6 blend films are located at 1.77 Å^−1^ (*d* = 3.54 Å) and 1.78 Å^−1^ (*d* = 3.52 Å), along with a CCL of 15.28 and 16.15 Å, respectively. JSM‐based films showcase tighter *π*–*π* stacking distances as compared with that (*d* = 3.61 Å) of PM6:Y6 [24].

Atomic force microscopy (AFM) was carried out to further study the nanoscale morphology of active layer surfaces. A more intermixed surface can be observed in JSM6‐based active layers, while large features can be found in JSM5‐based blend films, resulting in different root‐mean‐square (R_q_) values of 3.55 and 1.02 nm for JSM5 and JSM6 binary films (Figure 4d,e), respectively. We believe that the stronger S⋯N intermolecular interactions of JSM5 resulting from weaker end group interactions are likely the contributor to the large phase segregation that can be discerned from the recorded AFM images. This finding is consistent with TAS results, as such phase segregation cannot support efficient exciton migration and charge transfer, because it increases the distance that excitons must travel to reach the D:A interface. The corresponding longer exciton migration lifetime could result in a higher possibility of exciton recombination, which would also account for the lower performance of JSM5‐based devices.

The cost of the active layer material is a critical parameter in determining the prospects for OSC commercialization and market competitiveness. In this section, the costs of cutting‐edge FREAs, Y6, and BTP‐eC9, are used for comparison. A comparative analysis for synthesis costs of FREAs is conducted between JSM5 and Y6, which feature fluorine atoms on the end groups, known as IC‐2F, as well as between JSM6 and BTP‐eC9, having chlorine atoms in their end groups, known as IC‐2Cl. *C*_kg_ [22] (cost per kilogram) is employed to quantify the synthesis costs across the entire synthetic route (Schemes S1–S4 and Tables S17–S20) [47, 48]. The primary starting materials are the same for the four FREAs: 3‐bromothiophene, 4,5‐dichlorophthalic acid, and 1,2‐diaminobenzene. Among these, 3‐bromothiophene and 1,2‐diaminobenzene are used to construct the main molecular backbone, and 4,5‐dichlorophthalic acid is employed for the synthesis of end groups.

**FIGURE 5:**
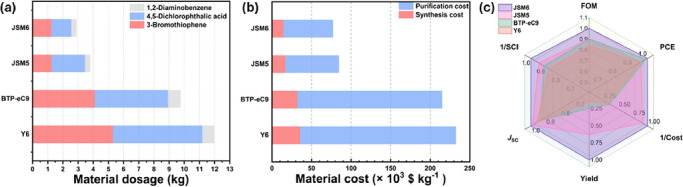
(a) Dosage bar chart of primary starting materials. (b) Synthesis cost bar chart for Y6, BTP‐eC9, JSM5, and JSM6. (c) Normalized radar chart benchmarking JSM5 and JSM6 against Y6 and BTP‐eC9 across six key indicators: FOM, PCE, 1/Cost, Yield, *J*_SC_, and 1/SCI.

JSMs share the same number of synthetic steps as their counterparts. However, the consumption of the three starting materials for JSM5 and JSM6 is significantly lower than that of β‐alkylated Y6 and BTP‐eC9 (Figure 5a). The reasons behind this are illustrated as follows: (I) In the final Knoevenagel condensation, the additional intermolecular interactions from β‐side chains of Y6 and BTP‐eC9 cores can lead to strong self‐packing and the formation of nanodomains in solution, resulting in a low‐yield reaction [49, 50]. The use of large excess amounts of end groups for promoting yields is contrary to the use of only 3 eq. in case of synthesizing JSM5 and JSM6 in high yields, surpassing 80% for the final product. As a result, only 1.31 kg of 4,5‐dichlorophthalic acid was consumed to produce end groups required for making 1 kg of JSM6, which is significantly lower than the 4.81 kg required for BTP‐eC9 [22]. Furthermore, the synthesis of IC‐2F requires one more step to convert chlorine atoms to fluorine atoms involving the precursor of IC‐2Cl, leading to higher consumption of 4,5‐dichlorophthalic acid (2.20 kg). (II) In the second last step, the aldehydes were introduced to the central cores through the Vilsmeier–Haack reaction, delivering a high yield of 81%, which is 17% higher than that of Y6 and BTP‐eC9 obtained by using LDA and DMF at low temperatures [2, 3, 23]. (III) Due to the simplified synthetic procedures, the synthesis of thienothiophene requires two fewer purification steps than that of the β‐alkylated counterparts. In addition, the total yield for synthesizing thienothiophene is 68%, which is much higher than the 38% yield for β‐alkylated counterparts [51, 52]. This results in a significant reduction in the amount of 3‐bromothiophene required for the synthesis of JSM5 and JSM6. Collectively, the steps involved in the preparation of β‐alkylated components determine the total consumption of three starting materials. Consequently, the total yields of JSMs are 3 times higher than those of Y6 and BTP‐eC9 (Table S22). The *C*_kg_ values associated with the synthesis of Y6, BTP‐eC9, JSM5, and JSM6 are summed to be 232, 212, 84, and 77 × 10^3^ USD kg^−1^, respectively (Figure 5b). Notably, JSM6 has the lowest material cost, and both JSMs exhibit approximately one‐third of the material costs compared to Y6 and BTP‐eC9, indicating that JSM6 is one of the cheapest FREAs reported to date.

We further calculated the figure of merit (FOM) to estimate the cost‐efficiency balance for each FREA: FOM = PCE/SCI, where SCI is the synthetic complexity index that quantifies the practical effort required to synthesize the molecule (e.g., step count, yields, and purification burden) [1, 21, 53]. The SCI covers five parameters: the number of synthetic steps (NSS), reciprocal yields (RY) decided by the total synthetic yields, the number of unit operations for the purification of the comonomers (NUO), the number of column chromatography (NCC), and the number of hazardous chemicals used in the preparations (NHC) (Table S21). Y6 exhibited the highest values of NSS, RY, and NUO, contributing to a maximum SCI of 97.6%, resulting in the lowest FOM value of 17.6. In contrast, JSM6, with the lowest synthetic complexity index and the highest PCE, attained the highest FOM value of 20.9, offering great potential for OSC commercialization (Table S22). In addition, JSMs exhibit comparable SCI compared to the high‐performance NFREA, 2BTh‐2F‐C_2_ [21]. The normalized radar plot (Figure 5c) provides a comprehensive overall view of the relationship between molecular manufacturability (Yield, FOM, 1/SCI, and 1/Cost) and device performance (PCE and *J*_SC_). Notably, JSM6 exhibited superior values in Yield, FOM, PCE, 1/SCI, *J*_SC,_ and 1/Cost, enclosing the largest overall area among the four acceptors. This result emphasizes that the β‐side‐chain‐free design narrows the cost‐efficiency gap by delivering high photocurrent and efficiency without the complicated purification from β‐alkylated Y‐series molecules.

## 3 Conclusion

In conclusion, we designed and synthesized two β‐side‐chain‐free FREAs, JSM5 and JSM6, featuring thienothiophene moieties in the backbone and fluorinated or chlorinated end groups. We systematically studied the optoelectronic and molecular packing properties from the comparison with β‐alkylated Y6. GIWAXS and single‐crystal analysis reveal that JSMs display tighter *π*–*π* molecular packing than Y6. Furthermore, we uncovered an unusual tetrameric structure, where molecules from the same plane have parallel S⋯N intermolecular interactions between different molecules originating from the elimination of β‐side chains. This kind of structure leverages γ‐shaped dimer conformations and acts as a repeating unit to form a compact layer‐by‐layer 3D‐network with superior intermolecular connectivity for efficient charge transport, which is also supported by its higher electron mobility measured by SCLC in neat films as compared to Y6. Notably, JSM5 molecules with less restriction from end groups possess well‐aligned N⋯S‐N angles and orbital overlap in the same plane, whereas those of JSM6 are mitigated due to stronger end group interactions from IC‐2Cl, exhibiting a trade‐off between these two interactions. The indicated stronger local S⋯N intermolecular interactions of JSM5 could be the underlying reason for unfavorable phase segregation. PM6:JSM6‐based devices deliver the champion average PCE of 17.8% (PCE_max_ = 18.0%), with a *V*_OC_ of 0.809 V, a high *J*_SC_ of 28.0 mA cm^−2,^ and an FF of 0.797, while JSM5 delivers a lower average PCE of 16.8%. Despite a lower *V*_OC_, the high *J*_SC_ enables JSM6 to achieve higher PCEs than its β‐alkylated counterpart BTP‐eC9 under the same conditions. Furthermore, the calculations of *C*_kg_ (cost per kilogram) and FOM (Figure of Merit) metrics demonstrate that JSM6 is among the most cost‐effective FREAs reported, while maintaining a comparable SCI relative to other high‐performance NFREAs. These results demonstrate that β‐side‐chain‐free FREAs establish a new design framework that combines high performance with low‐cost and offers a promising pathway for scaling up acceptor materials.

## Author Contributions

J.Z. and Y.W. contributed equally to this work.

## Conflicts of Interest

The authors declare no conflicts of interest.

## Supporting information




**Supporting File**: advs75738‐sup‐0001‐SuppMat.docx.

## Data Availability

The data that support the findings of this study are available from the corresponding author upon reasonable request.

## References

[1] Y. Cui , H. Yao , J. Zhang , et al., “Over 16% Efficiency Organic Photovoltaic Cells Enabled by a Chlorinated Acceptor With Increased Open‐Circuit Voltages,” Nature Communications 10 (2019): 2515, 10.1038/s41467-019-10351-5. PMC6555805 31175276

[2] J. Yuan , Y. Zhang , L. Zhou , et al., “Single‐Junction Organic Solar Cell With Over 15% Efficiency Using Fused‐Ring Acceptor With Electron‐Deficient Core,” Joule 3 (2019): 1140–1151, 10.1016/j.joule.2019.01.004.

[3] Y. Cui , H. Yao , J. Zhang , et al., “Single‐Junction Organic Photovoltaic Cells With Approaching 18% Efficiency,” Advanced Materials 32 (2020): 1908205, 10.1002/adma.201908205. 32227399

[4] S. Liu , J. Yuan , W. Deng , et al., “High‐Efficiency Organic Solar Cells With Low Non‐radiative Recombination Loss and Low Energetic Disorder,” Nature Photonics 14 (2020): 300–305, 10.1038/s41566-019-0573-5.

[5] Z. Gao , Q. Chen , M. Duan , et al., “Resolving the Efficiency‐Mechanical Trade‐off in Organic Solar Cells: 20.4% Efficiency by Hydrogen‐bonding Engineering,” Angewandte Chemie International Edition 138 (2026): 24211, 10.1002/anie.202524211. 41606433

[6] X. Dai , B. Fan , X. Xu , and Q. Peng , “Synergistic Interface Engineering via o‐Difluorobenzene‐Mediated HPWO Crystallization and ITO Fluorination for 20.57% Efficiency Organic Solar Cells,” Advanced Materials 37 (2025): 2503072, 10.1002/adma.202503072. 40277195

[7] C. Li , M. Deng , H. Chen , et al., “A Halogenated Volatile Additive Strategy for Regulating Crystallization Kinetics and Enabling 20.40% Efficiency Polymer Solar Cells With Low Non‐Radiative Recombination Energy Loss,” Energy & Environmental Science 18 (2025): 5564–5576, 10.1039/D5EE01368B.

[8] W. Shi , Q. Han , W. Zhao , et al., “A Large Conjugated Rigid Dimer Acceptor Enables 20.19% Efficiency in Organic Solar Cells,” Energy & Environmental Science 18 (2025): 5356–5364, 10.1039/D5EE00878F.

[9] Y. Ding , W. A. Memon , S. Xiong , et al., “Flexible‐Linked Oligomeric Acceptors: Precise Synthesis and Enhanced Photovoltaic/Mechanical Properties for Stretchable Devices,” Angewandte Chemie International Edition 64 (2025): 202502596, 10.1002/anie.202502596. 40461760

[10] N. Yang , S. Zhang , Y. Cui , J. Wang , S. Cheng , and J. Hou , “Molecular Design for Low‐cost Organic Photovoltaic Materials,” Nature Reviews Materials 10 (2025): 404–424, 10.1038/s41578-025-00792-4.

[11] C. Li , X. Zhang , N. Yu , et al., “Simple Nonfused‐Ring Electron Acceptors With Noncovalently Conformational Locks for Low‐Cost and High‐Performance Organic Solar Cells Enabled by End‐Group Engineering,” Advanced Functional Materials 32 (2022): 2108861, 10.1002/adfm.202108861.

[12] X. Gu , R. Zeng , Y. Hou , et al., “Precisely Regulating Intermolecular Interactions and Molecular Packing of Nonfused‐Ring Electron Acceptors via Halogen Transposition for High‐Performance Organic Solar Cells,” Angewandte Chemie International Edition 63 (2024): 202407355, 10.1002/anie.202407355. 38837587

[13] X. Zhang , L. Qin , J. Yu , et al., “High‐Performance Noncovalently Fused‐Ring Electron Acceptors for Organic Solar Cells Enabled by Noncovalent Intramolecular Interactions and End‐Group Engineering,” Angewandte Chemie International Edition 60 (2024): 12475–12481, 10.1002/anie.202100390. 33749088

[14] P. Jiang , Y. Liu , J. Song , and Z. Bo , “Emergence of Low‐Cost and High‐Performance Nonfused Ring Electron Acceptors,” Accounts of Chemical Research 57 (2024): 3419–3432, 10.1021/acs.accounts.4c00592. 39567220

[15] G. Chai , Y. Chang , Z. Peng , et al., “Enhanced Hindrance From Phenyl Outer Side Chains on Nonfullerene Acceptor Enables Unprecedented Simultaneous Enhancement in Organic Solar Cell Performances With 16.7% Efficiency,” Nano Energy 76 (2020): 105087, 10.1016/j.nanoen.2020.105087.

[16] G. Chai , Y. Chang , J. Zhang , et al., “Fine‐Tuning of Side‐chain Orientations on Nonfullerene Acceptors Enables Organic Solar Cells With 17.7% Efficiency,” Energy & Environmental Science 14 (2021): 3469–3479, 10.1039/D0EE03506H.

[17] Y. Chang , J. Zhang , Y. Chen , et al., “Achieving Efficient Ternary Organic Solar Cells Using Structurally Similar Non‐Fullerene Acceptors With Varying Flanking Side Chains,” Advanced Energy Materials 11 (2021): 2100079, 10.1002/aenm.202100079.

[18] B. Fan , W. Gao , X. Wu , et al., “Importance of Structural Hinderance in Performance–Stability Equilibrium of Organic Photovoltaics,” Nature Communications 13 (2022): 5946, 10.1038/s41467-022-33754-3. PMC9547926 36209165

[19] J. Liang , M. Pan , Z. Wang , et al., “Branched Alkoxy Side Chain Enables High‐Performance Non‐Fullerene Acceptors With High Open‐Circuit Voltage and Highly Ordered Molecular Packing,” Chemistry of Materials 34 (2022): 2059–2068, 10.1021/acs.chemmater.1c03311.

[20] C. Li , J. Zhou , J. Song , et al., “Non‐fullerene Acceptors With Branched Side Chains and Improved Molecular Packing to Exceed 18% Efficiency in Organic Solar Cells,” Nature Energy 6 (2021): 605–613, 10.1038/s41560-021-00820-x.

[21] R. Zeng , M. Zhang , X. Wang , et al., “Achieving 19% Efficiency in Non‐fused Ring Electron Acceptor Solar Cells via Solubility Control of Donor and Acceptor Crystallization,” Nature Energy 9 (2024): 1117–1128, 10.1038/s41560-024-01564-0.

[22] X. Kong , N. Yang , X. Zhang , et al., “Suppressed Non‐radiative Loss and Efficient Hole Transfer at a Small Highest Occupied Molecular Orbital Offset Endows Binary Organic Solar Cells With 19.73% Efficiency and a Small Efficiency‐Cost Gap,” Energy & Environmental Science 18 (2025): 386–396, 10.1039/D4EE03000A.

[23] D. Yuk , M. H. Jee , C. W. Koh , et al., “Simplified Y6‐Based Nonfullerene Acceptors: in‐Depth Study on Molecular Structure‐Property Relation, Molecular Dynamics Simulation, and Charge Dynamics,” Small 19 (2023): 2206547, 10.1002/smll.202206547. 36541782

[24] G. Zhang , X. Chen , J. Xiao , et al., “Delocalization of Exciton and Electron Wavefunction in Non‐fullerene Acceptor Molecules Enables Efficient Organic Solar Cells,” Nature Communications 11 (2020): 3943, 10.1038/s41467-020-17867-1. PMC7414148 32770068

[25] H. Liang , H. Chen , P. Wang , et al., “Molecular Packing and Dielectric Property Optimization Through Peripheral Halogen Swapping Enables Binary Organic Solar Cells With an Efficiency of 18.77%,” Advanced Functional Materials 33 (2023): 2301573, 10.1002/adfm.202301573.

[26] Y. Sun , L. Wang , C. Guo , et al., “π‐Extended Nonfullerene Acceptor for Compressed Molecular Packing in Organic Solar Cells To Achieve Over 20% Efficiency,” Journal of the American Chemical Society 146 (2024): 12011–12019, 10.1021/jacs.4c01503. 38639467

[27] T. Liu , Y. Zhang , Y. Shao , et al., “Asymmetric Acceptors With Fluorine and Chlorine Substitution for Organic Solar Cells Toward 16.83% Efficiency,” Advanced Functional Materials 30 (2020): 2000456, 10.1002/adfm.202000456.

[28] S. Li , L. Zhan , Y. Jin , et al., “Asymmetric Electron Acceptors for High‐Efficiency and Low‐Energy‐Loss Organic Photovoltaics,” Advanced Materials 32 (2020): 2001160, 10.1002/adma.202001160. 32390241

[29] Z. Luo , R. Ma , Z. Chen , et al., “Altering the Positions of Chlorine and Bromine Substitution on the End Group Enables High‐Performance Acceptor and Efficient Organic Solar Cells,” Advanced Energy Materials 10 (2020): 2002649, 10.1002/aenm.202002649.

[30] D. Mo , H. Chen , J. Zhou , et al., “Isomeric Effects of Chlorinated End Groups on Efficient Solar Conversion,” Journal of Materials Chemistry A 8 (2020): 23955–23964, 10.1039/D0TA09306H.

[31] W. Shockley and H. J. Queisser , “Detailed Balance Limit of Efficiency of p‐n Junction Solar Cells,” Journal of Applied Physics 32 (1961): 510–519, 10.1063/1.1736034.

[32] T. Stergiopoulos and F. Polycarpos , “Minimizing Energy Losses in Dye‐Sensitized Solar Cells Using Coordination Compounds as Alternative Redox Mediators Coupled With Appropriate Organic Dyes,” Advanced Energy Materials 2 (2012): 616–627, 10.1002/aenm.201100781.

[33] G. Li , X. Zhang , L. O. Jones , et al., “Systematic Merging of Nonfullerene Acceptor π‑Extension and Tetrafluorination Strategies Affords Polymer Solar Cells With >16% Efficiency,” Journal of the American Chemical Society 143 (2021): 6123–6139, 10.1021/jacs.1c00211. 33848146

[34] G. P. Kini , S. J. Jeon , and D. K. Moon , “Design Principles and Synergistic Effects of Chlorination on a Conjugated Backbone for Efficient Organic Photovoltaics: a Critical Review,” Advanced Materials 32 (2020): 1906175, 10.1002/adma.201906175. 32020712

[35] F. R. Lin , K. Jiang , W. Kaminsky , Z. Zhu , and A. K.‐Y. Jen , “A Non‐fullerene Acceptor With Enhanced Intermolecular π‑Core Interaction for High‐Performance Organic Solar Cells,” Journal of the American Chemical Society 142 (2020): 15246–15251, 10.1021/jacs.0c07083. 32830487

[36] W. Zhu , A. P. Spencer , S. Mukherjee , et al., “Crystallography, Morphology, Electronic Structure, and Transport in Non‐Fullerene/Non‐Indacenodithienothiophene Polymer:Y6 Solar Cells,” Journal of the American Chemical Society 142 (2020): 14532–14547, 10.1021/jacs.0c05560. 32698577

[37] C. He , Z. Chen , T. Wang , et al., “Asymmetric Electron Acceptor Enables Highly Luminescent Organic Solar Cells With Certified Efficiency Over 18%,” Nature Communications 13, no. 1 (2022): 2598, 10.1038/s41467-022-30225-7. PMC9095617 35545620

[38] G. Kupgan , X.‐K. Chen , and J. L. Brédas , “Molecular Packing of Non‐fullerene Acceptors for Organic Solar Cells: Distinctive Local Morphology in Y6 vs. ITIC Derivatives,” Materials Today Advances 11 (2021): 100154, 10.1016/j.mtadv.2021.100154.

[39] V. Coropceanu , J. Cornil , D. A. S. Filho , Y. Olivier , R. Silbey , and J.‐L. Brédas , “Charge Transport in Organic Semiconductors,” Chemical Reviews 107 (2007): 926–952, 10.1021/cr050140x. 17378615

[40] J. L. Brédas , D. Beljonne , V. Cornopean , and J. Cornil , “Charge‐Transfer and Energy‐Transfer Processes in π‐Conjugated Oligomers and Polymers: a Molecular Picture,” Chemical Reviews 104 (2004): 4971–5003, 10.1021/cr040084k. 15535639

[41] V. Coropceanu , X.‐K. Chen , T. Wang , Z. Zheng , and J.‐L. Brédas , “Charge‐Transfer Electronic States in Organic Solar Cells,” Nature Reviews Materials 4 (2019): 689–707, 10.1038/s41578-019-0137-9.

[42] B. Savoie , K. Kohlstedt , N. Jackson , et al., “Mesoscale Molecular Network Formation in Amorphous Organic Materials,” Proceedings of the National Academy of Sciences 111 (2014): 10055–10060, 10.1073/pnas.1409514111. PMC4104918 24982179

[43] P. Li and Z.‐H. Lu , “Charge‐Transport Processes in Host–Dopant Organic Semiconductors,” Advanced Electronic Materials 6 (2020): 1901147, 10.1002/aelm.201901147.

[44] M. Stolterfoht , A. Armin , B. Philippa , et al., “Photocarrier Drift Distance in Organic Solar Cells and Photodetectors,” Scientific Reports 5 (2015): 9949, 10.1038/srep09949. 25919439 PMC4412075

[45] T.‐K. Chu and O.‐K. Song , “Apparent Thickness Dependence of Mobility in Organic Thin Films Analyzed by Gaussian Disorder Model,” Journal of Applied Physics 104 (2008): 023711, 10.1063/1.2959825.

[46] Y. Che , M. R. Niazi , R. Izquierdo , and D. F. Perepichka , “Mechanism of the Photodegradation of A‐D‐A Acceptors for Organic Photovoltaics**,” Angewandte Chemie International Edition 60 (2021): 24833–24837, 10.1002/anie.202109357. 34506067

[47] X. Wu , X. Zhang , J. Zhang , et al., “19.36% Efficiency Organic Solar Cells Based on Low‐Cost Terpolymer Donors With Simple Molecular Structures,” Advanced Functional Materials 34 (2024): 2405168, 10.1002/adfm.202405168.

[48] Z. Li , X. Zhang , X. Kong , et al., “Ultrafast Charge Transfer and Suppressed Non‐radiative Energy Loss Enabled by Trifluoromethyl‐substituted Low‐cost Polymer Donors for Efficient Organic Solar Cells,” Science China Chemistry 68 (2025): 3797–3806, 10.1007/s11426-024-2563-0.

[49] Y.‐J. Xue , Z.‐Y. Lai , H.‐C. Lu , et al., “Unraveling the Structure−Property−Performance Relationships of Fused‐Ring Nonfullerene Acceptors: Toward a C‑Shaped orthoBenzodipyrrole‐Based Acceptor for Highly Efficient Organic Photovoltaics,” Journal of the American Chemical Society 146 (2023): 833–848, 10.1021/jacs.3c11062. 38113458

[50] M. Gao , K. Zhang , W. Zhao , et al., “Variable‐Temperature X‐Ray Scattering Unveils the Solution Aggregation Structures and Processing Resiliency of High‐Efficiency Organic Photovoltaics With Iodinated Electron Acceptors,” Advanced Materials 37 (2025): 02275, 10.1002/adma.202502275. 40776815

[51] K. Kawabata , M. Takeguchi , and H. Goto , “Optical Activity of Heteroaromatic Conjugated Polymer Films Prepared by Asymmetric Electrochemical Polymerization in Cholesteric Liquid Crystals: Structural Function for Chiral Induction,” Macromolecules 46 (2013): 2078–2091, 10.1021/ma400302j.

[52] W. L. Liau , Y. J. Su , H. F. Tseng , J. T. Chen , and C. S. Hsu , “Synthesis and Characterisation of Liquid Crystal Molecules Based on Thieno [3,2‐b] Thiophene and Their Application in Organic Field‐Effect Transistors,” Liquid Crystals 44 (2017): 557–565, 10.1080/02678292.2016.1225835.

[53] R. Po , G. Bianchi , C. Carbonera , and A. Pellegrino , ““All That Glisters Is Not Gold”: An Analysis of the Synthetic Complexity of Efficient Polymer Donors for Polymer Solar Cells,” Macromolecules 48 (2015): 453–461, 10.1021/ma501894w.

[54] T. M. McPhillips , S. E. McPhillips , H.‐J. Chiu , et al., “Blu‐Ice and the DistributedControlSystem: Software for Data Acquisition and Instrument Control at Macromolecular Crystallography Beamlines,” Journal of Synchrotron Radiation 9 (2002): 401–406, 10.1107/S0909049502015170. 12409628

[55] D. Aragao , J. Aishima , H. Cherukuvada , et al., “MX_2_: A High‐flux Undulator Microfocus Beamline Serving both the Chemical and Macromolecular Crystallography Communities at the Australian Synchrotron,” Journal of Synchrotron Radiation 25 (2018): 885–891, 10.1107/S1600577518003120. 29714201 PMC5929359

