# Supplementary material for: Side Chain Elimination Enables Low‐Cost Fused‐Ring Acceptors and Reveals a Compact Tetrameric Structure for High‐Photocurrent Organic Solar Cells
Source: Adv Sci (Weinh). 2026 May 19;13(45):e75738. doi: 10.1002/advs.75738 (PMC13336129; doi:10.1002/advs.75738)
Supplement: Supplementary file 1 [file ADVS-13-e75738-s001.docx]

**Supporting information**

**Side Chain Elimination Enables Low-Cost Fused-Ring Acceptors and Reveals a Compact Tetrameric Structure for High-Photocurrent Organic Solar Cells**

Jinhui Zhao, Yi Wu, Witold M. Bloch, Caroline V.I. Andersson, Leandro R. Franco, Rafael B. Ribeiro, Chuangcheng Hong, Wei Zhang, Xun Pan, Joost Kimpel, Christian Müller, Zhicai He,* Bin Zhang,* Ergang Wang,* Mats R. Andersson*

**1. Methodology**

**1.1 Fabrication of optimal devices**

The devices were fabricated with the following structure: ITO/PEDOT: PSS/active layer/PDINN/Ag. ITO substrates were cleaned sequentially in an ultrasonic bath using isopropyl alcohol, Milli-Q water, acetone, and again isopropyl alcohol, each for 15 minutes, and then dried in an oven at 80 °C for 12 hours. A layer of PEDOT: PSS was spin-coated onto the substrate surface at 4000 rpm for 30 s and thermally annealed at 150 °C for 15 min in ambient; the corresponding film thickness was approximately 30 nm. The PEDOT: PSS-coated substrates were then transferred into a nitrogen-filled glove box. The active layer solution was prepared through dissolving a weight ratio of 1:1.2 for PM6: JSMs mixture at a total concentration of 15 mg mL^-1^ in a chloroform (CF)/1,3,5-trichlorobenzene (TCB) solution, where 10 mg mL^-1^ of TCB was added as an additive. The active layer was deposited by spin-coating 16 μL of the blend solution at 3000 rpm for 35 s, yielding a film thickness of about 110 nm. The film was subsequently annealed at 100 °C for 5 min. Then, a methanol solution of PDINN (1.0 mg mL^-1^) was spin-coated at the top the active layer at 3000 rpm for 30 s to form the cathode buffer layer. Finally, Ag electrodes were thermally evaporated onto the devices through a shadow mask under a pressure of approximately 10^-4^ Pa. The active area of the device was 5.76 mm^2^, and the mask area was 4.00 mm^2^. The current density–voltage (*J*–*V*) characteristics of the devices were measured under illumination from an AM 1.5G solar simulator (Newport, 100 mW cm^-2^) using a Keithley 2420 Source Measure Unit. The external quantum efficiency (EQE) was characterized using a certified Newport IPCE measurement system (Model 66902). Hole mobilities were evaluated using hole-only devices with the structure ITO/PEDOT: PSS/active layer/MoO_3_/Ag, while electron mobilities were determined from electron-only devices with the structure ITO/ZnO/active layer/PDINN/Ag.

**1.2 GIWAXS**

GIWAXS patterns were obtained from Xeuss 3.0 SAXS/WAXS, with a detector of Eiger2R 1M. The test environment is in vacuum, at 300 K. The incident angle is 0.18°. The distance between detector and sample is 100 mm, and the X-ray wavelength is 1.54189 Å, with corresponding beam energy of 8.05 keV. The samples were spin-coated on a Si substrate.

**1.3 Single-crystal growth and measurement**

The single crystals of JSM5 and JSM6 were raised by a ternary solvent diffusion method. A solution of acceptor (1 mg/ 0.3 mL CF) was transferred into an NMR tube, followed by carefully covering a small amount of DCM (0.1 mL). Then the system was sealed by excessive methanol to the top. The tubes were left in the dark for two weeks until the color of the solutions had faded. The single crystals were placed in paratone-N oil, mounted on plastic loops, and flash-frozen in liquid nitrogen. The crystals were stored at cryogenic temperatures within dry shippers and transported to The Australian Synchrotron. X-ray diffraction data was collected at 100(2) K on the MX-2 beamline.^[1]^ Structures were solved by direct methods using SHELXT^[2]^ and refined with SHELXL^[3]^ and ShelXle^[4]^ as a graphical user interface. All non-hydrogen atoms were refined anisotropically and hydrogen atoms were included as invariants at geometrically estimated positions. X-ray experimental data is given in Table S1. Where appropriate, a GRADE dictionary for SHELXL was applied. This file contains target values and standard deviations for 1,2-distances (DFIX) and 1,3-distances (DANG), as well as restraints for planar groups (FLAT). As described below, the SQUEEZE routine was applied to the data.

**1.3.1 Specific details for JSM5**

The alkyl-substituted pyrrole rings in the backbone of the molecule were grouped into residues to address the atoms of repeating structural fragments with a single command. Stereochemical restraints for this moiety (residue JSM) were generated by the GRADE program using the GRADE Web Server (https://grade.globalphasing.org) and applied in the refinement. The refinement of ADP's was enabled by a combination of similarity restraints (SIMU) and rigid bond restraints (RIGU). These files are embedded in the CIF. The contribution of the electron density from disordered counterions and water solvent molecules, which could not be modeled with discrete atomic positions was handled using the SQUEEZE routine in PLATON. The solvent mask file (.fab) computed by PLATON was included in the SHELXL refinement via the ABIN instruction. 2017 electrons comprised the solvent mask, which equates to 263.4 per chemical formula (Z = 8). The removed electrons were assigned to 3 chloroform and 5 methanol molecules. The atoms from these molecules were updated in the UNIT formula before final refinement.

Figure S1: Asymmetric unit of JSM5 with probability ellipsoids at 50%. Hydrogen atoms omitted for clarity.

**1.3.2 Specific details for JSM6**

The alkyl-substituted pyrrole rings in the backbone of the molecule were grouped into residues to address the atoms of repeating structural fragments with a single command. Stereochemical restraints for this moiety (residue JSM) were generated by the GRADE program using the GRADE Web Server (https://grade.globalphasing.org) and applied in the refinement. Modelling the alkyl chain C10/C8 moiety however resulted in an unstable refinement, due to a lack of electron density in the F_obs_ map for the full length of these chains. A better data set could not be obtained with other samples and crystallisation methods. This reflects the flexibility of the alkyl chains and the solvent dependent nature of the crystals. To achieve stable refinement, the alkyl chains were truncated. The remaining carbon atoms were well represented in the Fobs map (detail in Fig. S3). The refinement of ADP's was enabled by a combination of similarity restraints (SIMU) and rigid bond restraints (RIGU). These files are embedded in the CIF. The contribution of the electron density from disordered counterions and water solvent molecules, which could not be modelled with discrete atomic positions was handled using the SQUEEZE routine in PLATON. The solvent mask file (.fab) computed by PLATON was included in the SHELXL refinement via the ABIN instruction. 161 electrons comprised the solvent mask, which equates to 161 per chemical formula (Z = 1). The removed electrons were assigned to 2 chloroform and 2 methanol molecules. The atoms from these molecules were updated in the UNIT formula before final refinement.

Figure S2: Asymmetric unit of JSM6 with probability ellipsoids at 50%. Hydrogen atoms omitted for clarity.

Figure S3: F_obs_ map of the four crystallographically independent molecules of JSM6 that crystallise as part of the asymmetric unit. The alkyl chains were truncated. The original length was C10/C8 (as expected from the synthesis of the molecule), and this was truncated to C9-C8/C8-C5, depending on the electron density. This compromise created stable refinement.

**1.4 Femtosecond transient absorption**

Femtosecond transient absorption measurements were carried out on a Harpia-TA spectroscopy system (Light Conversion), equipped with a light source from a femtosecond laser (Pharos, Light Conversion) operating at a central wavelength of 1030 nm, with pulse repetition rate of 100 kHz and pulse width of ~ 190 fs. The fundamental laser output was split into two beams: one was used to drive an optical parametric amplifier (Orpheus-HP, Light Conversion) to generate the pump beam for the Harpia-TA system, while the other was directed into the spectrometer to produce a supercontinuum white light serving as the probe beam. The relative time delays between the pump and probe beams were modulated using a mechanical delay stage. All measurements were conducted at room temperature. ^[5]^

**1.5 Cost feasibility calculation**

The synthesis cost of materials is greatly related to the preparation scale. The larger scale corresponds to the cheaper cost. Note that *C*_kg_ is a lab-scale synthetic cost metric, not a full module. Device cost estimate is not included. In this section, we calculated the amount of each starting materials, intermediates and reagents by deducing the synthesis of 1 kg of target acceptors through the step yield. The calculation is based on affordable scale and multiplied by the corresponding factor. The synthetic route of Y6 referred to the literature and the calculation for BTP-eC9 referred to the reported work.^[6]^ To keep good alignment in calculation with reported work, the quotations of all materials were all from Chinese chemical suppliers.

We referred to the reported framework to calculate the purification cost of each step. ^[7,8]^ We performed synthesis experiments according to literature and standard lab techniques. Four operations were involved in the experiments: quenching, extraction, column chromatography, and recrystallization. **Quenching:** in low-temperature reactions involving LDA and n-BuLi, they were quenched by water to finalize the reaction. In other cases, NaOH and 10M HCl solution were used to neutralize the reaction at a certain stage. **Extraction:** the amount of solvent for extraction varied due to different molecular weight. The larger weight the molecule has, the more solvent is used. The amount 100 mL × 3 was used to extract 15 g of intermediate at the early stage of synthesis. The amount of solvent increased to 30 mL × 3 for extracting 500 mg of fused ring structures. **Column chromatography:** 200-300 mesh silica gel was utilized for purification. The weight ratio of silica gel and material varied from 200:1 to 800:1, depending on the molecular weight, polarity, solubility and by-products. The polarity of eluent referred to literature and the volume ratio of eluent and silica gel (45 g/ 100 mL) varied from 4:1 to 12:1. **Recrystallization:** The recrystallization operation for intermediates referred to the literature. 300 mL of CF and 100 MeOH were used to recrystallize 600 mg of JSM5 or JSM6 in the last step. In addition, the costs of water, MgSO_4_ and sand were ignored due to their low price and dosage.

**1.6 FOM calculation**

The calculation referred to the equation: FOM = PCE/SCI. The synthetic complexity index (SCI) was calculated according to the equation as follows:^[9]^

SCI= 35 × NSS/NSS_max_ + 25 × log (RY/RY_max_) + 15 × NUO/NUO_max_ +15 × NCC/NCC_max_ + 10 × NHC/NHC_max_

where NSS = 1+ Σ (number of synthetic steps), RY = Σ (100/ total yield of the comonomers), NUO = Σ (number of the operations for reaction and corresponding purification, including quenching/neutralizing, extraction, column chromatography, recrystallization and distillation/sublimation), NCC = Σ(number of column chromatography purifications), NHC = Σ(number of the hazardous chemicals utilized in the synthesis according to CE Regulation n.1272/2008).^[8]^

**1.7 Computational Modelling Details**

**1.7.1 Redox potentials**

Electronic structure calculations for JSM5 and JSM6 were performed via Density Functional Theory (DFT) and Time-Dependent Density Functional Theory (TD-DFT) formalisms. For all calculations except the electronic couplings, the Gaussian 16 software^[10]^ was used with the screened range-separated hybrid (SRSH) LC-𝜔HPBE exchange-correlation functional.^[11]^ It features the Coulomb-attenuating method (CAM) partitioning of the electrostatic potential,^[12]^ i.e.,

$\frac{1}{r}=\frac{\alpha+\beta\cdot\text{erf}\left( \omega r \right)}{r}+\frac{1-\left( \alpha+\beta\cdot\text{erf}\left( \omega r \right) \right)}{r}$,

where $\alpha$, $\beta$ and $\omega$ are parameters that control the relation between short- and long-range contributions. A couple of approaches have been proposed to fit these constants,^[13,14]^ and we followed the common strategy of setting $\alpha$ = 0.2 to reduce DFT self-interaction error.^[15-18]^ Then, to guarantee the correct asymptotic decay of the Coulomb interaction,^[13]^ $\beta$ was chosen such that $\alpha+\beta=\epsilon^{-1}$, where $\epsilon$ is the static dielectric constant of the material. In contrast, $\omega$ is determined from gas-phase calculations. Given the similarity between JSM5/JSM6 and Y6/Y7 and the fact that $\omega$ mostly depends on the system size,^[19-20]^ we adopted the optimized value for Y6 of $\omega$ = 0.981 $a_{0}^{-1}$from our previous work.^[21]^ Considering the experimental $\epsilon$ of 3.3 for Y6^[22]^ and the expected higher values for chlorinated derivatives,^[23]^ $\beta$ was set as 0.1030 and 0.0564 for JSM5 and JSM6, as summarized in Table S1.

Table S1**.** Optimized set of parameters from LC-$\omega$HPBE exchange-correlation functional for JSM5, JSM6 and Y6.

| Acceptor | $\boldsymbol{\epsilon}$ | $\boldsymbol{\alpha}$ | $\boldsymbol{\beta}$ | $\boldsymbol{\omega}$ ($\boldsymbol{a}_{\boldsymbol{0}}^{\boldsymbol{-1}}$) |
| --- | --- | --- | --- | --- |
| JSM5 | 3.3**^22^** | 0.2 | 0.1030 | 0.981 |
| JSM6 | 3.9 | 0.2 | 0.0564 | 0.981 |
| Y6 | 3.3^22^ | 0.2 | 0.1030 | 0.981 |

For geometry optimizations and frequency calculations, the 6-311G(d,p) triple-$\zeta$ atomic basis set was employed, and an additional set of diffuse functions on heavy atoms was included via 6-311+G(d,p) basis set for computing the electronic energies and excited states. Environmental effects were described according to the Integral Equation Formalism (IEFPCM) variant^[24]^ of the Polarizable Continuum Model (PCM),^[25]^ using the Y6/Y7 static dielectric constants presented above and the Y6 dynamic dielectric constant of 5.3.^[22]^

Gibbs free energies used to compute the redox potentials were determined as the sum of gas-phase electronic energies ($E_{elec}$) with thermal contributions ($G_{trv}^{T}$) and the solvation free energy ($\Delta G_{solv}^{T}$),

$G = E_{elec}+G_{trv}^{T}+\Delta G_{solv}^{T}$ .

Thermal contributions were computed within the rigid-rotor harmonic oscillator (RRHO) model^[26]^ while the solvation free energy comes from the implicit solvent model using the thin-film dielectric constants. As a result, we can estimate the oxidation ($E_{oxi}$) and reduction ($E_{red}$) potentials, which correspond to the energy levels experimentally measured, via,

$E_{red}=G\left( M \right)-G\left( M^{-} \right)$ and $E_{oxi}=G\left( M \right)-G\left( M^{+} \right)$ ,

where $G\left( M \right)$, $G\left( M^{-} \right)$ and $G\left( M^{+} \right)$ are the Gibbs free energies of molecule $M$when neutral, with charge$-e$ or$+e$, respectively.

**1.7.2 UV-vis absorption spectra**

Using the same level of theory described in Sec. 1.1, and employing the corresponding optimized α, β, and γ parameters, we performed Time-Dependent Density Functional Theory (TDDFT) calculations at the ground-state optimized geometry of each molecule. Vertical excitation energies and oscillator strengths were computed for the lowest 50 singlet excited states. The UV–vis absorption spectra were then constructed by convoluting these discrete transitions with Gaussian line shapes of 0.05 eV width, using the excitation energies as peak positions and the oscillator strengths as intensities.

**1.7.3 Reorganization energies**

The reorganization energy is split into two contributions: one related to the surrounding medium ($\lambda_{s}$) and the other to the molecular degrees of freedom ($\lambda_{in}$). Considering that outer contributions are typically smaller in comparison to inner ones,^[27,28]^ we follow the common approach of neglecting $\lambda_{s}$,^[29,30]^ while $\lambda_{in}$ is determined according to Nelsen's four-point method^[31^**^]^** for both electrons and holes,

$\lambda_{in}^{h}=\left( E_{0}^{+} -E_{+} \right)+\left( E_{+}^{0}-E_{0} \right)$ and $\lambda_{in}^{e}=\left( E_{0}^{-}-E_{-} \right)+\left( E_{-}^{0}-E_{0} \right)$ ,

with superscripts indicating the charge of the system and subscripts indicating the optimized geometry, e.g., $E_{+}^{0}$ is the energy of the neutral at the ground-state geometry of the cation (with $+e$ charge).

**1.7.4 Marcus theory**

To investigate the charge-transport properties of the JSM5 and JSM6 crystals, we conducted a cluster analysis^[32]^ on the experimental crystal structures to identify the most representative dimeric arrangements within the lattice. Considering that the transfer integral is inversely proportional to the distance between acceptors, we only considered dimers with atoms from different acceptors within 5 Angstrom. Subsequently, the electronic couplings between the molecular fragments of these dimers were calculated using the ADF program package,^[33]^ through its *Transfer Integral* methodology.

In this approach, the Kohn-Sham Hamiltonian and overlap matrix elements are evaluated in the basis of fragment molecular orbitals, enabling the quantification of hole and electron transfer pathways. The effective electronic coupling, $V$, is obtained via a Löwdin‑type orthogonalization of the fragment orbitals, using

$V=\frac{J-S\left( e_{1}+e_{2} \right)/2}{1-S^{2}}$ ,

where J is the Hamiltonian matrix element, S the orbital overlap, and e₁ and e₂ the fragment site energies, as defined below:

$$J=\left\langle\phi_{1} | H_{KS} | \phi_{2} \right\rangle$$

$$S=\left\langle\phi_{1} | \phi_{2} \right\rangle$$

$$e_{1}=\left\langle\phi_{1} | H_{KS} | \phi_{1} \right\rangle$$

$$e_{2}=\left\langle\phi_{2} | H_{KS} | \phi_{2} \right\rangle$$

For electron mobility, $\phi_{1}$ and $\phi_{2}$ correspond to the LUMOs of fragments 1 and 2, respectively; for hole mobility, they correspond to the HOMOs. In these calculations, we adopted the pure PBE functional^[34]^ and the triple-zeta quality basis set TZP^[35]^ in accordance with previous work.^[36]^

From the transfer rates, we estimated the mobility of charge carriers using the Eistein relation, given by,^[37]^

$\mu_{e/h}=\frac{eD_{e/h}}{k_{B}T}$ ,

where $e$is the elementary charge and $D$the diffusion coefficient. The latter is described within a random-walk approximation, i.e., $D_{e/h}\approx L^{2}k_{e/h}$, with $L$corresponding to the separation between sites. Note that this approach typically yields an upper limit for mobilities.

**1.7.5 Clustering analysis**

From the experimental crystallographic data, we generated a 2x2x2 supercell (without side chains) and extracted all dimers with a maximum separation of 5 Å. Note that, by considering multiple unitary cells, we can also account for interfaces between molecules from different cells. As a result, we selected 306, 257 and 157 dimers of JSM5, JSM6 and Y6 molecules, respectively.

To identify unique motifs, we employed a hierarchical clustering procedure using the clusttraj package.^[32]^ The Hungarian method was employed to find the optimal atomic labeling, along with the Ward linkage scheme based on a minimized RMSD dissimilarity metric. Considering the crystalline pattern and the high planarity of these Y-shaped molecules, we respectively reduced the JSM5, JSM6 and Y6 dimers into 5, 7, and 5 unique subsets of interfaces (Table S5) with different relative populations (Table S7). The medoid clusters were selected as representative configurations and used to determine the transfer integrals, as described in Sec. 1.4.

**1.7.6 Graph-based analysis of crystal connectivity**

All graph analyses were performed in Python using MDAnalysis^[38]^ to handle the crystal structures and NetworkX^[39]^ for graph construction and analysis. Molecular crystals were taken from the experimental structures, i.e., the 2x2x2 supercells generated from experimental CIF files. Atoms were grouped into molecular fragments using bond connectivity inferred with the MDAnalysis toolkit, and each fragment was treated as a network node. Periodic boundary conditions were accounted for by using the unit-cell dimensions and the minimum-image convention for triclinic boxes. For every pair of molecular fragments $i$ and $j$, we computed the minimum intermolecular atom-atom distance ($d_{i,j}^{min}$), and defined an undirected edge between i and j whenever $d_{i,j}^{min}\leq r_{c}$, where $r_{c}$ is the distance cutoff. In practice, we first constructed a symmetric matrix of minimum distances $d_{i,j}^{min}$ for all fragment pairs, and then generated, for each chosen cutoff (here scanned from 3 to 20 Å in 1 Å steps), a simple undirected graph $G\left( r_{c} \right)$ by adding edges for all pairs satisfying this criterion.

For each graph $G\left( r_{c} \right)$, we computed the degree $z_{i}$ (number of neighbors within the cutoff) of each molecule and analyzed the resulting degree distribution. We extracted the average degree $\left\langle z_{r_{c}} \right\rangle$ and standard deviation $\sigma\left( z_{r_{c}} \right)$, as well as the fraction of molecules that exhibit the modal degree. These quantities provide a compact description of the density and heterogeneity of the local contact environments for a given cutoff. In addition, we identified all connected components of $G\left( r_{c} \right)$, recording for each cutoff the number of components, the component sizes, and the population fraction associated with each component. This allows us to track how the intermolecular contact network evolves from a highly fragmented set of small clusters to a fully percolated network (a single dominant component containing essentially all molecules). All statistics were collected for the three crystals (JSM5, JSM6 and Y6) under identical settings, and the numpy,^[40]^ pandas,^[41]^ and matplotlib^[42]^ libraries were also used to process the data and generate graphics.

**2. Synthesis and characterizations**

Materials and reagents were purchased from Sigma Aldrich and Alfa Chemical Co. Ltd, Meryer Co. Ltd, Solarmer, Inc and Energy Chemical Co. Ltd. The solubility of JSM5, JSM6 and Y6 are 26.4 mg/mL, 20.9 mg/mL and 26.7 mg/mL, respectively.

a) Quenching/neutralization; b) Extraction; c) Column chromatography; d) Recrystallization.

Scheme S1. The synthetic route of JSM5. Numbers correspond to chemicals in Table S17.

*Synthesis of JSM5*

The dialdehyde core 52 (70 mg, 0.066 mmol), 2-(5,6-difluoro-3-oxo-2,3-dihydro-1H-inden-1-ylidene) malononitrile (46 mg, 0.199 mmol), 0.02 ml pyridine and 15 ml Chloroform were added in a 100 ml round bottom flask. After degassing 3 times with Nitrogen, the mixture refluxed overnight. The mixture was precipitated in methanol and collected through filtration. The collected solid was purified by silica gel chromatography with Hexane:DCM = 1:1 to afford dark solid. Then the solid recrystallized in MeOH/CF to afford dark red crystals. (80 mg, yield: 86%). ^1^H-NMR (CDCl_3_, 600 MHz): δ (ppm) 9.02 (s, 2H), 8.57 (dd, 2H), 8.23 (s, 2H), 7.72 (t, 2H), 4.78 (d, 4H), 2.11 (dt, 2H), 1.23 - 0.97 (m, 64H), 0.80 (t, 12H).

a) Quenching/neutralization; b) Extraction; c) Column chromatography; d) Recrystallization.

Scheme S2. The synthetic route of JSM6. Numbers correspond to chemicals in Table S18.

*Synthesis of JSM6*

The dialdehyde core 47 (70 mg, 0.066 mmol), 2-(5,6-dichloro-3-oxo-2,3-dihydro-1H-inden-1-ylidene) malononitrile (52 mg, 0.199 mmol), 0.02 ml pyridine and 15 ml Chloroform were added in a 100 ml round bottom flask. After degassing 3 times with Nitrogen, the mixture refluxed overnight. The mixture was precipitated in methanol and collected through filtration. The collected solid was purified by silica gel chromatography with Hexane:DCM = 1:1.1 to afford dark solid. Then the solid recrystallized in MeOH/CF to afford dark red crystals. (77 mg, yield: 82%). ^1^H-NMR (CDCl_3_, 600 MHz): δ (ppm) 9.06 (s, 2H), 8.83 (s, 2H), 8.25 (s, 2H), 8.04 (s, 2H), 4.84 (d, 4H), 2.18 (dt, 2H), 1.25 – 1.04 (m, 64H), 0.84 (t, 12H).

a) Quenching/neutralization; b) Extraction; c) Column chromatography; d) Recrystallization; e) Distillation/sublimation.

Scheme S3. The synthetic route of Y6. Numbers correspond to chemicals in Table S21.

a) Quenching/neutralization; b) Extraction; c) Column chromatography; d) Recrystallization.

Scheme S4. The synthetic route of BTP-eC9. Numbers correspond to chemicals in Table S20.^[6]^

Figure S4. ^1^H-NMR spectrum of JSM5.

Figure S5. ^1^H-NMR spectrum of JSM6.

Figure S6. High Resolution Mass spectra of (a) JSM5 and (b) JMS6.

Figure S7**.** (a) Normalized UV-vis absorption spectra of both acceptors in solution and HOMO (bottom) and LUMO (top) isosurfaces for (b) JSM5 and (c) JSM6. The spectra were determined from the first 50 singlet excited states, considering Gaussian profiles with a standard deviation of 0.05 eV. Molecular orbitals are shown using the isovalue of 0.01.

Table S2. DFT calculation results of electronic properties for JSM5, JSM6 and Y6.

| Acceptor | $\boldsymbol{\lambda}_{\boldsymbol{max}}^{\boldsymbol{sol}}$ (nm) | $\boldsymbol{E}_{\boldsymbol{LUMO}}$  (eV) | $\boldsymbol{E}_{\boldsymbol{HOMO}}$  (eV) | $\boldsymbol{E}_{\boldsymbol{gap}}$  (eV) | $\boldsymbol{\lambda}_{\boldsymbol{in}}^{\boldsymbol{e}}$  (eV) | $\boldsymbol{\lambda}_{\boldsymbol{in}}^{\boldsymbol{h}}$  (eV) |
| --- | --- | --- | --- | --- | --- | --- |
| JSM5 | 730 | -3.86 | -5.81 | 1.95 | 0.128 | 0.178 |
| JSM6 | 740 | -3.93 | -5.74 | 1.80 | 0.114 | 0.172 |
| Y6 | 726 | -3.79 | -5.79 | 2.00 | 0.126 | 0.175 |

Figure S8. CV plots for JSM5, JSM6, Y6 and PM6 in solid state and Fc/Fc^+^ plot. Energy level = -e ($\varphi$_ox/re_ + 4.80) (eV). Using glassy carbon as working electrode, a Pt wire as counter electrode, and an Ag/Ag^+^ reference electrode calibrated using ferrocene/ferrocenium (Fc/Fc^+^) redox couple.

Figure S9. GIWAXS pattern of pure Y6 film.^[43]^

Table S3. The crystallographic data of JSM5 and JSM6.

| Compound | **JSM5** | **JSM6** |
| --- | --- | --- |
| CCDC number | 2484471 | 2484472 |
| Empirical formula | C_176_H_203_Cl_9_F_8_N_16_O_9_S_10_ | C_627_H_629_Cl_47_N_64_O_18_S_40_ |
| Formula weight | 3478.18 | 12297.46 |
| Crystal system | Orthorhombic | Triclinic |
| Space group | C222**_1_** | P-1 |
| *a (Å)* | 30.254(6) | 25.673(5) |
| *b (Å)* | 32.209(6) | 25.916(5) |
| *c* (Å) | 36.551(7) | 26.818(5) |
| α (º) | 90 | 78.17 |
| β (º) | 90 | 73.36 |
| γ (º) | 90 | 86.48 |
| Volume (Å^3^) | 35618(12) | 16733(7) |
| *Z* | 8 | 1 |
| Density (calc.) (Mg/mm^3^) | 1.297 | 1.220 |
| Absorption coefficient (mm^-1^) | 0.328 | 0.374 |
| F(000) | 14624 | 6422 |
| Crystal size (mm^3^) | 0.32 x 0.28 x 0.15 | 0.33 x 0.28 x 0.22 |
| θ range for data collection (º) | 0.923 to 24.718 | 0.803 to 24.713 |
| Reflections collected | 193871 | 192246 |
| Observed reflections [R(int)] | 29978 [0.0615] | 53608 [0.0347] |
| Goodness-of-fit on F^2^ | 1.384 | 1.348 |
| R_1_ [I>2σ(I)] | 0.1245 | 0.1135 |
| wR_2_ (all data) | 0.3467 | 0.3712 |
| Largest diff. peak and hole (e.Å-3) | 0.986 and -0.411 | 1.201 and -0.555 |
| Data / restraints / parameters | 29978 / 4770 / 2338 | 53608 / 6026 / 3550 |

Figure S10. Representative structures extracted from unit cells of (a) JSM5, (b) JSM6 and (c) Y6 crystals.

Table S4. Parameters of conformers.

| Conformer | | S⋯O distance (Å) | | Torsion angle D-A (^o^) | | Torsion angle  π -core (^o^) |
| --- | --- | --- | --- | --- | --- | --- |
|  |  | left | right | left | right |  |
| JSM5 | Left | 2.69 | 2.73 | 0.64 | 2.12 | 10.25 |
|  | Right | 2.68 | 2.64 | 0.49 | 0.42 | 22.52 |
| JSM6 | Up | 2.68 | 2.60 | 3.23 | 2.69 | 39.80 |
|  | Down | 2.66 | 2.66 | 1.52 | 5.66 | 23.51 |
|  | Left | 2.67 | 2.66 | 3.42 | 0.46 | 30.46 |
|  | Right | 2.65 | 2.67 | 4.61 | 3.80 | 0.84 |
| Y6 | Left | 2.71 | 2.69 | 8.75 | 4.49 | 9.81 |
|  | Right | 2.64 | 2.62 | 2.02 | 5.02 | 1.48 |

Figure S11. Side views of tetrameric structures for JSM5 (a) and (c); JSM6 (b) and (d).

Figure S12. Dimeric conformations of (a) JSM5, (b) JSM6 and (c) Y6.

Figure S13. S⋯N interaction of (a) JSM5 and (b) JSM6; N⋯S-N angles of (c) JSM5 and (d) JSM6; bond length within (e) JSM5 and (f) JSM6; S⋯N distances of (g) JSM5 and (h) JSM6.

Table S5. Relative populations (in percentage) of $i$-shaped dimer interfaces within a 2x2x2 supercell. Only interfaces with atoms from different molecules separated by at maximum of 5 Angstrom were considered.

| Acceptor | S | M | C | O | $\boldsymbol{\gamma}$ | X | Others |
| --- | --- | --- | --- | --- | --- | --- | --- |
| JSM5 | 20.9 | 7.8 | 0.0 | 9.8 | 41.8 | 19.6 | 0.0 |
| JSM6 | 18.7 | 9.3 | 15.6 | 5.8 | 31.1 | 12.4 | 7.0 |
| Y6 | 22.3 | 0.0 | 15.3 | 0.0 | 22.9 | 8.9 | 30.6 |

Table S6. Dimeric single crystal packing structures and the calculated electron and hole transfer couplings for JSM5, JSM6 and Y6.

| \|V\| (meV)/ JSM5 | X | S | $\boldsymbol{\gamma}$ | M | O |  |  |
| --- | --- | --- | --- | --- | --- | --- | --- |
| Electron transfer | 0.95 | 22.82 | **36.41** | 30.00 | 0.13 |  |  |
| Hole transfer | 2.06 | 29.40 | **30.83** | 38.40 | 0.01 |  |  |
|  |  |  |  |  |  |  |  |
| \|V\| (meV)/ JSM6 | X | C | $\boldsymbol{\gamma}$ | M | O | S | L |
| Electron transfer | 1.28 | 62.30 | **27.27** | 43.20 | 12.12 | 18.68 | 0.42 |
| Hole transfer | 2.03 | 33.78 | **29.94** | 27.49 | 23.07 | 0.63 | 0.13 |
|  |  |  |  |  |  |  |  |
| \|V\| (meV)/ Y6 | S1 | S2 | S3 | C | $\boldsymbol{\gamma}$ | m | CC |
| Electron transfer | 47.52 | 30.34 | 0.10 | 67.99 | **64.47** | 0.25 | 0.25 |
| Hole transfer | 13.81 | 2.81 | 0.07 | 16.14 | **61.84** | 0.04 | 0.04 |

Cluster analysis of the crystal packing reveals a small number of recurring dimer motifs, comprising both π-π stacked geometries (S-, C-, ϒ-, and M-shaped) with substantial co-facial overlap, and non π-π stacked arrangements (X- and L-shaped) characterized by offset contacts and minimal orbital interaction. These clusters capture the key packing environments and form the basis for comparing charge-transport pathways in the two acceptors. In JSM5 crystals, the non π-π stacked X- and O-shaped conformations display only negligible electron (from 0.13 to 0.95 meV), and hole (from 0.01 to 2.06 meV) couplings, whereas the π-π stacked S-, M- and $\gamma$-shaped dimers enhance these values by more than an order of magnitude, reaching up to 36 meV for electrons and 38 meV for holes. JSM6 follows the same structural trend but with substantially higher efficiencies for the electronic transport: its non-stacked X-shaped conformation again yields very weak couplings, while the π-π stacked C-, ϒ-, and M-shaped dimers generate significantly larger electronic (LUMO-LUMO) interactions, resulting in electron couplings of 27-62 meV, the strongest observed in either system. Notably, JSM6 exhibits a greater number of π-π stacked configurations, as its O-shaped conformation displays significant ending groups overlap, whereas the analogous O-shaped motif in JSM5 does not. The larger couplings of JSM6 are also related to the higher polarizability of chlorine, the formation of Cl-mediated intermolecular contacts (Cl⋯π and Cl⋯S conformational locks), and the resulting tighter π–π packing, which collectively facilitate both electron and hole transport relative to fluorine.^[43,44]^ Interestingly, the Y6 dimers follow the same trend as the β-chain-free acceptors but exhibit higher transfer integrals at the π-π stacked interfaces, due to the better overlap between the resonant structures. As a result, when considering the slight differences in reorganization energy (Table S2), the higher transfer integrals tend to dictate the overall charge mobility, as shown in Table S7.

Table S7. Charge transfer rates and mobilities for electrons and holes in JSM5, JSM6 and Y6 dimers.

| Molecule | Dimer | $\boldsymbol{k}_{\boldsymbol{e}}$ (Hz) | $\boldsymbol{k}_{\boldsymbol{h}}$ (Hz) | $\boldsymbol{\mu}_{\boldsymbol{e}}$ (cm^2^·V^-1^·s^-1^) | $\boldsymbol{\mu}_{\boldsymbol{h}}$ (cm^2^·V^-1^·s^-1^) |
| --- | --- | --- | --- | --- | --- |
| JSM5 | S  Y  M  X  O | 2.25E+12  5.73E+12  3.89E+12  3.90E+09  7.30E+07 | 1.95E+12  2.15E+12  3.33E+12  9.59E+09  2.26E+05 | 2.41E+00  3.04E+00  4.48E+00  3.79E-03  7.74E-05 | 2.09E+00  1.14E+00  3.84E+00  9.32E-03  2.39E-07 |
| JSM6 | S  Y  M  X  O  C  L | 2.08E+09  3.90E+12  9.78E+12  8.59E+09  7.70E+11  2.03E+13  9.25E+08 | 1.40E+08  2.18E+12  1.84E+12  1.00E+10  1.30E+12  2.78E+12  4.12E+07 | 2.34E-03  2.09E+00  1.10E+01  8.34E-03  8.18E-02  1.49E+01  6.76E-04 | 1.58E-04  1.17E+00  2.07E+00  9.74E-03  1.38E-01  2.03E+00  3.01E-05 |
| Y6 | S-far  S-close  Y  X  X-not  C | 4.44E+07  1.00E+13  1.85E+13  2.55E+12  2.78E+08  2.05E+13 | 1.15E+07  4.47E+11  8.97E+12  8.28E+10  3.75E+06  6.11E+11 | 1.36E-04  1.37E+01  4.64E+00  1.87E+00  4.15E-04  1.29E+01 | 3.52E-05  6.10E-01  2.25E+00  6.05E-02  5.61E-06  3.84E-01 |

The critical role of molecular orbital overlap is even more pronounced when comparing the rates and mobilities. For instance, even the Y6 dimers with the same shape, the slightly higher separation between molecules yields mobilities up to 5 orders of magnitude lower, i.e., 1.37E+01 and 1.36E-04 cm^2^·V^-1^·s^-1^ electron mobilities for S1- and S3-shaped dimers, respectively. This high sensitivity raises concerns about the representativeness of medoids relative to the entire cluster and highlights the challenges of modelling conformational effects on charge transfer within the hopping regime. Nevertheless, we can propose a simplified approach to determine the effective charge mobility in crystalline films by considering a weighted average based on the population of each shape. The results are presented in Tab. S9.

Table S8. Average charge transfer rates and mobilities of JSM5, JSM6 and Y6 weighted by the population of each dimer shape.

| Molecule | $\boldsymbol{k}_{\boldsymbol{e}}$ (Hz) | $\boldsymbol{k}_{\boldsymbol{h}}$ (Hz) | $\boldsymbol{\mu}_{\boldsymbol{e}}$ (cm^2^·V^-1^·s^-1^) | $\boldsymbol{\mu}_{\boldsymbol{h}}$ (cm^2^·V^-1^·s^-1^) |
| --- | --- | --- | --- | --- |
| JSM5 | 3.17E+12 | 1.57E+12 | 2.13E+00 | 1.22E+00 |
| JSM6 | 5.34E+12 | 1.36E+12 | 4.00E+00 | 8.84E-01 |
| Y6 | 9.32E+12 | 2.23E+12 | 5.55E+00 | 6.86E-01 |

Table S9. Charge mobility of neat and blend films.

| D:A | *μ*_h_ (cm^2^ ·V^−1^ ·s^-1^) | *μ*_e_ (cm^2^ ·V^−1^ ·s^-1^) | *μ*_h_/ *μ*_e_ |
| --- | --- | --- | --- |
| JSM5 | / | 5.95 | / |
| JSM6 | / | 7.51 | / |
| Y6 | / | 4.81 | / |
| PM6:JSM5 | 6.09 × 10^-4^ | 6.77 × 10^-4^ | 0.9 |
| PM6:JSM6 | 9.80 × 10^-4^ | 9.15 × 10^-4^ | 1.1 |
| PM6:Y6 | 3.97 × 10^-4^ | 2.16 × 10^-4^ | 1.8 |

Table S10. Summary of component analysis for the graph-based crystalline networks of JSM5, JSM6 and Y6. We set a 3.0 Å cutoff for intermolecular distances (replacing side chains with methyl groups) in the three crystals, ensuring that only first neighbours with non-negligible transfer integrals are linked. The relative percentages correspond to the fraction of all molecules within one component with the corresponding size.

| Molecule | Number of molecules | Number of components | Number of unique components | Component size (relative percentage) | Number of components with this size |
| --- | --- | --- | --- | --- | --- |
| JSM5 | 160 | 28 | 4 | 36 (22.5%)  16 (10.0%)  9 (5.62%)  1 (0.62%) | 2  2  4  20 |
| JSM6 | 120 | 10 | 2 | 56 (46.67%)  1 (0.83%) | 2  8 |
| Y6 | 72 | 31 | 4 | 6 (8.33%)  4 (5.56%)  2 (2.78%)  1 (1.39%) | 5  4  4  18 |

Figure S14. Average degree for JSM5, JSM6 and Y6 crystals as a function of the cutoff distance between atoms from different molecules. Shaded areas consider the standard deviation. Each molecule corresponds to a node, and the cutoff distance to the threshold for establishing an edge between nodes.

Table S11. Photovoltaic performance parameters of the OSCs based on PM6:JSM5 with different annealing temperature, under the irradiation of AM 1.5G, 100 mW cm^-2^.

| Temperature  (°C) | *V_oc_*  [V] | *J_SC_*  [mA cm^-2^] | FF  [%] | PCE  [%] |
| --- | --- | --- | --- | --- |
| 75 | 0.794 | 27.22 | 76.78 | 16.60 |
| 100 | 0.793 | 27.44 | 78.94 | 17.18 |
| 125 | 0.805 | 27.10 | 76.88 | 16.77 |
| 150 | 0.802 | 26.86 | 76.30 | 16.43 |
| 175 | 0.781 | 25.66 | 76.10 | 15.25 |

Table S12. Photovoltaic performance parameters of the OSCs based on PM6:JSM5 with different annealing time (100 °C), under the irradiation of AM 1.5G, 100 mW cm^-2^.

| Time  (min) | *V_oc_*  [V] | *J_SC_*  [mA cm^-2^] | FF  [%] | PCE  [%] |
| --- | --- | --- | --- | --- |
| 5 | 0.793 | 27.59 | 78.94 | 17.27 |
| 10 | 0.791 | 26.49 | 76.27 | 15.98 |
| 15 | 0.761 | 25.79 | 75.02 | 14.72 |
| 20 | 0.744 | 24.75 | 73.61 | 13.55 |

Table S13. Photovoltaic performance parameters of the OSCs based on PM6:JSM6 with different annealing temperature, under the irradiation of AM 1.5G, 100 mW cm^-2^.

| Temperature  (°C) | *V_oc_*  [V] | *J_SC_*  [mA cm^-2^] | FF  [%] | PCE  [%] |
| --- | --- | --- | --- | --- |
| 75 | 0.797 | 27.47 | 78.75 | 17.24 |
| 100 | 0.795 | 27.69 | 80.28 | 17.67 |
| 125 | 0.796 | 27.19 | 79.68 | 17.25 |
| 150 | 0.799 | 26.86 | 78.24 | 16.43 |
| 175 | 0.784 | 26.57 | 76.95 | 16.02 |

Table S14. Photovoltaic performance parameters of the PSCs based on PM6:JSM6 with different annealing time (100 °C), under the irradiation of AM 1.5G, 100 mW cm^-2^.

| Time  (min) | *V_oc_*  [V] | *J_SC_*  [mA cm^-2^] | FF  [%] | PCE  [%] |
| --- | --- | --- | --- | --- |
| 5 | 0.809 | 27.98 | 79.70 | 18.05 |
| 10 | 0.809 | 26.90 | 79.91 | 17.39 |
| 15 | 0.803 | 27.61 | 77.87 | 17.27 |
| 20 | 0.797 | 27.72 | 76.20 | 16.84 |

Figure S15. (a) The UV-vis spectra of D/A blend film; (b) Efficiency-distribution histograms of the four devices.

Table S15. Parameters for hole transfer kinetics within optimal blend films.

| Blend film | Amplitude  A_1_ | Lifetime  τ_1_(ps) | Amplitude  A_2_ | Lifetime  τ_2_(ps) |
| --- | --- | --- | --- | --- |
| PM6:JSM5 | 0.61 | IRF | 0.39 | 0.8 |
| PM6:JSM6 | 0.49 | IRF | 0.49 | 0.7 |

Figure S16. The UV-vis spectra for photostability of (a) JSM5, (b) JSM6, (c) Y6 and (d) BTP-eC9 neat films exposed to 1 Sun (AM 1.5G, AAA solar simulator) in an ambient environment for 160 hours.

Table S16. 1D-GIWAXS parameters of blend films.

| Blend film | IP (Å ^-1^) | *d* (Å) | CCL (Å) | OOP (Å ^-1^) | *d* (Å) | CCL (Å) |
| --- | --- | --- | --- | --- | --- | --- |
| PM6:JSM5 | 0.30 | 20.94 | 41.6 | 1.77 | 3.54 | 15.28 |
| PM6:JSM6 | 0.27 | 23.27 | 37.7 | 1.78 | 3.52 | 16.15 |

Table S17. Material cost of synthesizing 1 kg of JSM5.

| **Starting Materials** | | | | |
| --- | --- | --- | --- | --- |
| **Number** | **Reagents** | **Unit price ($)** | **Dosage** | **Total price ($)** |
| **1** | 3-bromothiophene | 74.7/500 g | 1.26 kg | 188.3 |
| **2** | LDA 2M | 30.1/500 mL | 3.8 L | 228.8 |
| **3** | THF | 5.4/500 mL | 73.1 L | 789.5 |
| **4** | DMF | 4.0/500 mL | 20.0 L | 160.0 |
| **6** | 2-Sulfanyl acetate | 39.8/500 g | 881 g | 72.1 |
| **7** | K_2_CO_3_ | 4.0/500 g | 2.3 kg | 18.4 |
| **9** | LiOH·H_2_O | 29.1/500 g | 538 g | 31.3 |
| **10** | HCl | 2.8/500 mL | 1.6 L | 9.0 |
| **12** | Cu | 12.8/500 g | 252 g | 6.5 |
| **13** | Quinoline | 12.1/500 mL | 10.1 L | 244.4 |
| **15** | n-Butyllithium 2M | 22.7/500 mL | 2.6 L | 119.4 |
| **16** | Bu_3_SnCl | 64.1/500 g | 2.0 kg | 256.4 |
| **18** | 4,5-dichlorophthalic acid | 237.1/100 g | 2.2 kg | 5145 |
| **19** | Ac_2_O | 10.9/500 mL | 14.5 L | 316.1 |
| **21** | KF | 6.46/500 g | 11.6 kg | 149.9 |
| **22** | mPEG | 15.1/500 g | 145.9 g | 4.4 |
| **23** | Sulfolane | 16.0/500 mL | 5.8 L | 185.6 |
| **25** | Tert-butyl-acetoacetate | 21.6/500 g | 832 g | 35.9 |
| **26** | Et_3_N | 16.8/500 mL | 3.8 L | 127.7 |
| **28** | EtOH | 1.2/500 mL | 9.1 L | 21.8 |
| **29** | Malononitrile | 29.2/500 g | 400 g | 23.4 |
| **30** | CH_3_COONa | 5.4/500 g | 373 g | 4.0 |
| **32** | 2-Octyldodecan-1-ol | 24.3/500g | 2.15 kg | 104.5 |
| **33** | MeOH | 2.8/500 mL | 333.0 L | 1864.8 |
| **34** | Br_2_ | 21.0/500 g | 5.8 kg | 243.6 |
| **35** | PPh_3_ | 17.9/500 g | 4.1 kg | 146.8 |
| **37** | 1,2-Diaminobenzene | 17.8/500 g | 346 g | 12.3 |
| **38** | SOCl_2_ | 9.3/500 g | 789 g | 14.7 |
| **40** | HBr | 5.3/500 mL | 2.7 L | 28.6 |
| **42** | HNO_3_ | 3.2/500 mL | 502 g | 3.3 |
| **43** | CF_3_SO_3_H | 62.8/500 g | 4.9 kg | 615.4 |
| **45** | Pd(PPh_3_)_2_Cl_2_ | 2070.9/100 g | 163 g | 3375.6 |
| **47** | *o*-DCB | 7.0/500 mL | 12.6 L | 176.4 |
| **48** | KI | 48.4/500 g | 1.6 kg | 154.9 |
| **50** | POCl_3_ | 35.6/500 mL | 194 mL | 13.9 |
| **51** | 1,2-Dichloroethane | 8.1/500 mL | 38.8 L | 628.6 |
| **53** | CH_3_Cl | 5.7/500 mL | 82.9 L | 945.1 |
| **54** | Pyridine | 6.2/500 mL | 170 ml | 2.1 |
|  |  |  | **Sum: 16468.5 USD** | |
| **Purification process** | | | | |
| **55** | DCM | 2.8/500 mL | 3343.4 L | 18722.8 |
| **56** | EtOH | 1.2/500 mL | 10.0 L | 24.0 |
| **57** | NaOH | 5.3/500 g | 1.6 kg | 17.0 |
| **58** | HCl 10M | 2.8/500 mL | 3.5 L | 19.6 |
| **59** | Hexane Fraction | 1.8/500 mL | 5132.7 L | 18477.8 |
| **60** | EA | 1.8/500 mL | 62.4 L | 224.7 |
| **61** | Silica gel (200~300 mesh) | 9.0/500 g | 872.0 kg | 15696 |
| **62** | Ac_2_O | 10.9/500 mL | 233.3 L | 5086.7 |
| **63** | CH_3_Cl | 5.7/500 mL | 799.2 L | 9110.9 |
|  |  |  | **Sum: 67379.5 USD** | |
| **Total cost: 83848.0 USD** | | | | |

Table S18. Material cost of synthesizing 1 kg of JSM6.

| **Starting Materials** | | | | |
| --- | --- | --- | --- | --- |
| **Number** | **Reagents** | **Unit price ($)** | **Dosage** | **Total price ($)** |
| **1** | 3-bromothiophene | 74.7/500 g | 1.24 kg | 185.3 |
| **2** | LDA 2M | 30.1/500 mL | 3.8 L | 228.8 |
| **3** | THF | 5.4/500 mL | 73.4 L | 792.7 |
| **4** | DMF | 4.0/500 mL | 20.0 L | 160.0 |
| **6** | 2-Sulfanyl acetate | 39.8/500 g | 881 g | 70.1 |
| **7** | K_2_CO_3_ | 4.0/500 g | 2.33 | 18.6 |
| **9** | LiOH·H_2_O | 29.1/500 g | 538.9 g | 31.4 |
| **10** | HCl | 2.8/500 mL | 5.5 L | 30.8 |
| **12** | Cu | 12.8/500 g | 252 g | 6.4 |
| **13** | Quinoline | 12.1/500 mL | 10.1 L | 244.4 |
| **15** | n-Butyllithium 2M | 22.7/500 mL | 2.7 L | 122.6 |
| **16** | Bu_3_SnCl | 64.1/500 g | 2.0 kg | 256.4 |
| **18** | 4,5-dichlorophthalic acid | 237.1/100 g | 1.31 kg | 3106 |
| **19** | Ac_2_O | 10.9/500 mL | 12.1 L | 263.8 |
| **20** | Tert-butyl-acetoacetate | 21.6/500 g | 871 g | 37.6 |
| **21** | Et_3_N | 16.8/500 mL | 4.4 L | 148.2 |
| **23** | EtOH | 1.2/500 mL | 10.1 L | 21.8 |
| **24** | Malononitrile | 29.2/500 g | 394 g | 23.0 |
| **25** | CH_3_COONa | 5.4/500 g | 372 g | 4.1 |
| **27** | 2-Octyldodecan-1-ol | 24.3/500g | 1.3 kg | 63.2 |
| **28** | MeOH | 2.8/500 mL | 334.4 L | 1872.6 |
| **29** | Br_2_ | 21.0/500 g | 5.8 kg | 243.6 |
| **30** | PPh_3_ | 17.9/500 g | 4.1 kg | 146.8 |
| **32** | 1,2-Diaminobenzene | 17.8/500 g | 347 g | 12.3 |
| **33** | SOCl_2_ | 9.3/500 g | 791 g | 14.7 |
| **35** | HBr | 5.3/500 mL | 2.7 L | 28.6 |
| **37** | HNO_3_ | 3.2/500 mL | 0.5 kg | 3.3 |
| **38** | CF_3_SO_3_H | 62.8/500 g | 4.9 kg | 615.4 |
| **40** | Pd(PPh_3_)_2_Cl_2_ | 2070.9/100 g | 162 g | 3354.9 |
| **42** | KI | 48.4/500 g | 1.7 kg | 164.6 |
| **43** | *o*-DCB | 7.0/500 mL | 12.7 L | 177.8 |
| **45** | POCl_3_ | 35.6/500 mL | 195 ml | 13.9 |
| **46** | 1,2-Dichloroethane | 8.1/500 mL | 38.9 L | 630.2 |
| **48** | CH_3_Cl | 5.7/500 mL | 83.3 L | 949.6 |
| **49** | Pyridine | 6.2/500 mL | 186 mL | 2.3 |
|  |  |  | **Sum: 14045.8 USD** | |
| **Purification process** | | | | |
| **50** | DCM | 2.8/500 mL | 3185.7 L | 17840.0 |
| **51** | EtOH | 1.2/500 mL | 10.2 L | 24.3 |
| **52** | NaOH | 5.3/500 g | 1.6 kg | 131.9 |
| **53** | HCl 10M | 2.8/500 mL | 6.6 L | 36.9 |
| **54** | Hexane Fraction | 1.8/500 mL | 4757.4 L | 17126.6 |
| **55** | EA | 1.8/500 mL | 62.6 L | 225.4 |
| **56** | Silica gel (200~300 mesh) | 9.0/500 g | 851.3 kg | 15234.6 |
| **57** | CH_3_Cl | 5.7/500 mL | 802.9 L | 9153.1 |
| **58** | Ac_2_O | 10.9/500 mL | 140.1 L | 3020.2 |
|  |  |  | **Sum: 62827.0 USD** | |
| **Total cost: 76781.4 USD** | | | | |

Table S19. Material cost of synthesizing 1 kg of Y6.

| **Starting Materials** | | | | |
| --- | --- | --- | --- | --- |
| **Number** | **Reagents** | **Unit price ($)** | **Dosage** | **Total price ($)** |
| **1** | 3-bromothiophene | 74.7/500 g | 5.3 kg | 791.8 |
| **2** | Dodecanoyl chloride | 40.3/500 g | 7.1 kg | 572.3 |
| **3** | AlCl_3_ | 4.9/500 g | 4.3 kg | 42.1 |
| **4** | DCM | 2.8/500 mL | 253.8 L | 1421.3 |
| **6** | 2-Sulfanyl acetate | 39.8/500 g | 3.2 L | 254.7 |
| **7** | K_2_CO_3_ | 4.0/500 g | 8.0 kg | 64.0 |
| **8** | DMF | 4.0/500 ml | 83.7 L | 669.6 |
| **10** | EtOH | 1.2/500 mL | 91.9 L | 220.6 |
| **11** | NaOH | 5.3/500 g | 2.6 kg | 27.6 |
| **13** | Cu | 12.8/500 g | 1.2 kg | 30.7 |
| **14** | Quinoline | 12.1/500 mL | 57.6 L | 1393.9 |
| **16** | n-Butyllithium 2M | 22.7/500 mL | 8.0 L | 363.2 |
| **17** | Bu_3_SnCl | 64.1/500 g | 4.0 kg | 512.8 |
| **18** | THF | 5.4/500 ml | 122.1 L | 1318.7 |
| **20** | 4,5-dichlorophthalic acid | 237.1/100 g | 5.95 kg | 13983.0 |
| **21** | Ac_2_O | 10.9/500 mL | 39.7 L | 865.5 |
| **23** | KF | 6.46/500 g | 4.6 kg | 59.3 |
| **24** | mPEG | 15.1/500 g | 398 g | 12.0 |
| **25** | Sulfolane | 16.0/500 mL | 15.9 L | 540.8 |
| **27** | Tert-butyl-acetoacetate | 21.6/500 g | 2.3 kg | 99.4 |
| **28** | Et_3_N | 16.8/500 mL | 10.2 L | 342.7 |
| **30** | Malononitrile | 29.2/500 g | 1.1 kg | 64.2 |
| **31** | CH_3_COONa | 5.4/500 g | 1.0 kg | 10.8 |
| **32** | 2-Ethyl-hexanol | 9.2/500g | 1.4 kg | 25.8 |
| **34** | Br_2_ | 21.0/500 g | 19.8 kg | 831.6 |
| **35** | PPh_3_ | 17.9/500 g | 4.3 kg | 153.9 |
| **37** | 1,2-Diaminobenzene | 17.8/500 g | 831 g | 29.7 |
| **38** | SOCl_2_ | 9.3/500 g | 3.2 kg | 59.5 |
| **40** | HBr | 5.3/500 mL | 6.4 L | 67.8 |
| **42** | HNO_3_ | 3.2/500 mL | 1.2 L | 7.7 |
| **43** | CF_3_SO_3_H | 62.8/500 g | 11.8 kg | 1482.1 |
| **45** | Pd(PPh_3_)_2_Cl_2_ | 2070.9/100 g | 173 g | 3582.7 |
| **46** | Toluene | 2.8/500 mL | 27.9 L | 156.2 |
| **48** | P(OEt)_3_ | 10.8/500 ml | 18.1 L | 391.0 |
| **49** | *o*-DCB | 7.0/500 mL | 7.3 L | 102.2 |
| **50** | KOH | 4.8/500 g | 726 g | 7.0 |
| **53** | CH_3_Cl | 5.7/500 mL | 348.3 L | 3970.6 |
| **54** | Pyridine | 6.2/500 mL | 740 mL | 9.2 |
|  |  |  | **Sum: 34538.0 USD** | |
| **Purification process** | | | | |
| **55** | DCM | 2.8/500 mL | 6492.6 L | 36358.6 |
| **56** | EtOH | 1.2/500 mL | 99.8 L | 239.5 |
| **57** | NaOH | 5.3/500 g | 6.0 kg | 63.6 |
| **58** | HCl 10M | 2.8/500 mL | 25.1 L | 140.6 |
| **59** | Hexane Fraction | 1.8/500 mL | 859.0 L | 3092.4 |
| **60** | EA | 1.8/500 mL | 418.2 L | 1505.5 |
| **61** | Silica gel (200~300 mesh) | 9.0/500 g | 2919.9 kg | 52558.2 |
| **62** | MeOH | 1.3/500 mL | 412.0 L | 1030.0 |
| **63** | Ac_2_O | 10.9/500 L | 752.5 L | 16404.5 |
| **64** | Petroleum Ether | 2.8/500 mL | 15320.8 L | 85796.5 |
|  |  |  | **Sum: 197189.0 USD** | |
| **Total cost: 231727.0 USD** | | | | |

Table S20. Material cost of synthesizing 1 kg of BTP-eC9.^[6]^

| **Starting Materials** | | | | |
| --- | --- | --- | --- | --- |
| **Number** | **Reagents** | **Unit price ($)** | **Dosage** | **Total price ($)** |
| **1** | 3-bromothiophene | 74.7/500 g | 4.1 kg | 611.9 |
| **2** | 1-Chlorononane | 40.3/500 g | 4.8 kg | 388.8 |
| **3** | AlCl_3_ | 4.9/500 g | 3.4 kg | 32.7 |
| **4** | DCM | 2.8/500 mL | 168.9 L | 938.4 |
| **6** | 2-Sulfanyl acetate | 39.8/500 g | 2.5 L | 216.5 |
| **7** | K_2_CO_3_ | 4.0/500 g | 6.3 kg | 50.1 |
| **8** | DMF | 4.0/500 ml | 72.0 L | 575.9 |
| **10** | EtOH | 1.2/500 mL | 97.7 L | 217.2 |
| **11** | NaOH | 5.3/500 g | 1.8 kg | 19.2 |
| **13** | Cu | 12.8/500 g | 906.9 g | 23.1 |
| **14** | Quinoline | 12.1/500 mL | 45.4 L | 1091.9 |
| **16** | n-Butyllithium 2M | 22.7/500 mL | 6.73 L | 288.4 |
| **17** | Bu_3_SnCl | 64.1/500 g | 4.0 kg | 511.3 |
| **18** | THF | 5.4/500 ml | 122.8 L | 1296.1 |
| **20** | 4,5-dichlorophthalic acid | 237.1/100 g | 4.8 kg | 12708.4 |
| **21** | Ac_2_O | 10.9/500 mL | 21.7 L | 469.2 |
| **23** | Sulfolane | 16.0/500 mL | 16.2 L | 520.8 |
| **24** | Tert-butyl-acetoacetate | 21.6/500 g | 2.3 kg | 99.3 |
| **25** | Et_3_N | 16.8/500 mL | 4.7 L | 155.0 |
| **27** | Malononitrile | 29.2/500 g | 1.1 kg | 64.5 |
| **28** | CH_3_COONa | 5.4/500 g | 1.0 kg | 10.9 |
| **30** | 2-Butyloctan-1-ol | 160.8/500g | 1.1 kg | 342.8 |
| **31** | Br_2_ | 21.0/500 g | 17.5 kg | 729.7 |
| **32** | PPh_3_ | 17.9/500 g | 1.8 kg | 64.8 |
| **34** | Benzene-1,2-diamine | 17.8/500 g | 0.8 kg | 29.75 |
| **35** | SOCl_2_ | 9.3/500 g | 2964.8 kg | 54.6 |
| **37** | HBr | 5.3/500 mL | 26.3 L | 275.6 |
| **38** | HNO_3_ | 3.2/500 mL | 1.2 kg | 4.82 |
| **40** | CF_3_SO_3_H | 62.8/500 g | 11.7 kg | 1456.3 |
| **42** | Pd(PPh_3_)_2_Cl_2_ | 2070.9/100 g | 173.6 kg | 3579.9 |
| **43** | Toluene | 2.8/500 mL | 28.0 L | 155.6 |
| **45** | P(OEt)_3_ | 10.8/500 ml | 18.2 L | 377.7 |
| **46** | *o*-DCB | 7.0/500 mL | 7.3 L | 101.0 |
| **48** | KOH | 4.8/500 g | 727.4 g | 7.6 |
| **49** | CH_3_Cl | 5.7/500 mL | 324.1 L | 3605.1 |
| **50** | Pyridine | 6.2/500 mL | 7.2 L | 88.0 |
|  |  |  | **Sum: 31162.9 USD** | |
| **Purification process** | | | | |
| **51** | DCM | 2.8/500 mL | 5633.8 L | 31298.8 |
| **52** | EtOH | 1.2/500 mL | 194.0 L | 431.1 |
| **53** | NaOH | 5.3/500 g | 6.0 kg | 12.3 |
| **54** | HCl 10M | 2.8/500 mL | 750.5 L | 4169.3 |
| **55** | n-Hexane | 2.2/500 mL | 859.0 L | 429.5 |
| **56** | EA | 1.8/500 mL | 322.0 L | 1162.8 |
| **57** | Silica gel (200~300 mesh) | 9.0/500 g | 2822.1 kg | 50954.6 |
| **58** | MeOH | 1.3/500 mL | 324.1 L | 810.1 |
| **59** | Ac_2_O | 10.9/500 L | 603 L | 13054.9 |
| **60** | Petroleum Ether | 2.8/500 mL | 14096.9 | 78316.1 |
|  |  |  | **Sum: 180639.5 USD** | |
| **Total cost: 211802.4 USD** | | | | |

Table S21. RY and NUO parameters of acceptors.

| Acceptor | Steps | Yield (%) | RY | Number of purification process | | | | | |
| --- | --- | --- | --- | --- | --- | --- | --- | --- | --- |
|  |  |  |  | a) | b) | c) | d) | e) | NUO |
| Y6 | 18 | 0.85 | 117.64 | 9 | 11 | 9 | 3 | 1 | 33 |
| BTP-eC9 | 17 | 1.41 | 70.92 | 7 | 13 | 10 | 3 | 0 | 33 |
| JSM5 | 18 | 3.56 | 28.09 | 7 | 12 | 9 | 3 | 0 | 31 |
| JSM6 | 17 | 5.65 | 17.70 | 7 | 12 | 9 | 3 | 0 | 31 |
| 2BTh-2F-C_2_ | 17 | 3.65 | 27.40 | 7 | 10 | 9 | 2 | 0 | 28 |

^a)^ Quenching/neutralization; ^b)^ Extraction; ^c)^ Column chromatography; ^d)^ Recrystallization; ^e)^ Distillation/sublimation.

Table S22. Calculation of SC and FOM for acceptors.

| Acceptor | NSS | RY | NUO | NCC | NHC | SCI% | PCE% | FOM |
| --- | --- | --- | --- | --- | --- | --- | --- | --- |
| Y6 | 18 | 117.64 | 33 | 9 | 53 | 97.64 | 17.22 | 17.63 |
| BTP-eC9 | 17 | 70.92 | 33 | 10 | 51 | 94.19 | 17.87 | 18.97 |
| JSM5 | 18 | 28.09 | 31 | 9 | 58 | 90.73 | 17.27 | 19.03 |
| JSM6 | 17 | 17.70 | 31 | 9 | 55 | 85.85 | 18.04 | 21.01 |
| 2BTh-2F-C_2_ | 17 | 27.40 | 28 | 9 | 49 | 85.11 | 19.02 | 22.34 |

NSS_max_ = 18; RY_max_ = 117.64; NUO_max_ = 33; NCC_max_ = 10; NHC_max_ = 58.

**Reference**

[1] D. Aragao, J. Aishima, H. Cherukuvada, R. Clarken, M. Clift, N. P. Cowieson, D. J. Ericsson, C. L. Gee, S. Macedo, N. Mudie, S. Panjikar, J. R. Price, A. R. Tunnicliffe, R. Rostan, Rachel Williamson, T. T. C> Davies, “MX2: a high-flux undulator microfocus beamline serving both the chemical and macromolecular crystallography communities at the Australian Synchrotron” *J Synchrotron Radiat.* **2018**, *25*, 885-891.
[2] G. Sheldrick, “*SHELXT* – Integrated space-group and crystal-structure determination” *Foundation and Advances* **2015**, *71*, 3-8.
[3] G. Sheldrick, “Crystal structure refinement with SHELXL” *Structural Chemistry* **2015**, *71*, 3-8.
[4] C. [Hübschle](https://scripts.iucr.org/cgi-bin/citedin?search_on=name&author_name=H%26uuml%3Bbschle%2C%20C%2EB%2E), G. Sheldrick, B. Dittrich, “*ShelXle*: a Qt graphical user interface for *SHELXL*” *J. Appl. Crystallography* **2011**, *44*, 1281-1284.
[5] C. Wang, N. Zhao, H. Zhang, X. Zhang, X. Lin, H. Liu, F. Dang, W. Zhang, J. Sun, P. Chen, H. Chen, P. Han, P. Li, “Ultrawide UV to NIR Emission in Double Perovskite Nanocrystals via the Self-Trapping State Engineering Strategy” *ACS Sustainable Chem. Eng.* **2023**, *11*, 14659-14666.
[6] X. Kong, N. Yang, X. Zhang, J. Zhang, Z. Li, X. Li, Y. Wu, R. Sun, J. Liao, X. Li, J. Min, G. Yang, C. Kai, “Suppressed non-radiative loss and efficient hole transfer at a small highest occupied molecular orbital offset endows binary organic solar cells with 19.73% efficiency and a small efficiency-cost gap” *Energy Environ. Sci.* **2025**, *18*, 386.
[7] Y. Cui, H. Yao, J. Zhang, T. Zhang, Y. Wang, L. Hong, K. Xian, B. Xu, S. Zhang, J. Peng, Z. Wei, F. Gao, J. Hou, “Over 16% efficiency organic photovoltaic cells enabled by a chlorinated acceptor with increased open-circuit voltages” *Nat. Commun.* **2019**, *10*, 2515.
[8] J. Yuan, Y. Zhang, L. Zhou, G. Zhang, H.-L. Yip, T.-K. Lau, X. Lu, C. Zhu, H. Peng, P. A. Johnson, L. Mario, Y. Cao, U. Jacek, Y. Li, Y. Zou, “Single-Junction Organic Solar Cell with over 15% Efficiency Using Fused-Ring Acceptor with Electron-Deficient Core” *Joule* **2019**, *3*, 1140-1151.
[9] R. Po, G. Bianchi, C. Carbonera, A. Pellegrino, “All That Glisters Is Not Gold: An Analysis of the Synthetic Complexity of Efficient Polymer Donors for Polymer Solar Cells” *Macromolecules* **2015**, *48*, 453-461.
[10] M. J. Frisch, G. W. Trucks, H. B. Schlegel, G. E. Scuseria, M. A. Rob, J. R. Cheeseman, G. Scalmani, *Gaussian 16, Revision C.01*. **2016**.
[11] T. M. Henderson, A. F. Izmaylov, G. Scalmani, G. E. Scuseria, “Can short-range hybrids describe long-range-dependent properties?” *J. Chem. Phys*. **2009**, *131*, 044108.
[12] T. Yanai, D. Tew, N. Handy, “A new hybrid exchange-correlation functional using the Coulomb-attenuating method (CAM-B3LYP)” *Chem. Phys. Lett.* **2004**, *393*, 51-57.
[13] S. Bhandari, M. S. Cheung, E. Geva, L. Kronik, B. D. Dunietz, “Fundamental gaps of condensed-phase organic semiconductors from single-molecule calculations using polarization-consistent optimally tuned screened range-separated hybrid functionals” *J. Chem. Theory Comput.* **2018**, *14*, 6287–6294.
[14] L R. Franco, C. Marchiori, C. M. Araujo, “Unveiling the impact of exchange-correlation functionals on the description of key electronic properties of non-fullerene acceptors in organic photovoltaics” *J. Chem. Phys.* **2023**, *159*, 204110.
[15] D. A. Egger, S. Weissman, A. S. Refaely, S. Sharifzadeh, M. Dauth, R. Baer, S. Kümmel, J. B. Neaton, E. Zojer, L. Kronik, “Outer-valence electron spectra of prototypical aromatic heterocycles from an optimally tuned range-separated hybrid functional” *J. Chem. Theory Comput.* **2014**, *10*, 1934–1952.
[16] A. S. Refaely, S. Sharifzadeh, N. Govind, J. Autschbach, J. B. Neaton, R. Baer, L. Kronik, “Quasiparticle spectra from a nonempirical optimally tuned range-separated hybrid density functional” *Phys. Rev. Lett.* **2012**, *109*, 226405.
[17] D. Lüftner, A. S. Refaely, M. Pachler, R. Resel, M. G. Ramsey, L. Kronik, P. Puschnig, “Experimental and theoretical electronic structure of quinacridone” *Phys. Rev. B*. **2014**, *90*, 075204.
[18] J. Autschbach, M. Srebro, “Delocalization error and ‘functional tuning’ in Kohn–Sham calculations of molecular properties” *Acc. Chem. Res.* **2014**, *47*, 2592–2602.
[19] L. Kronik, T. Stein, A. S. Refaely, R. Baer, “Excitation Gaps of Finite-Sized Systems from Optimally Tuned Range-Separated Hybrid Functionals” *J. Chem. Theory Comput.* **2012**, *8*, 1515–1531.
[20] T. Körzdörfer, J. S. Sears, C. Sutton, J.-L. Brédas, “Long-range corrected hybrid functionals for π-conjugated systems: Dependence of the range-separation parameter on conjugation length” *J. Chem. Phys.* **2011**, *135*, 204107.
[21] J. Wu, F. Sun, X. Xia, L. R. Franco, Q. Chen, Y. Fu, R. B. Ribeiro, X. Lu, C. M. Araujo, X. Wang, R. Qiang, X. Guo, D. Yu, M. Zhang, E. Wang, “Over 18% Efficiency from Halogen-Free Solvent-Processed Polymer Solar Cells Enabled by Asymmetric Small Molecule Acceptors with Fluoro-Thienyl Extended Terminal” *Adv. Funct. Mater.* **2025**, *24*, 2423137.
[22] R. Kerremans, C. Kaiser, W. Li, N. Zarrabi, P. Meredith, A. Armin, “The Optical Constants of Solution-Processed Semiconductors—New Challenges with Perovskites and Non-Fullerene Acceptors” *Adv. Opt. Mater.* **2020**, *8*, 2000319.
[23] Y.-B. Wang, Y-B. et al. Effect of halogen/chalcogen substitution on the dielectric constant of asymmetric acceptor alloy to improve the efficiency and stability of inverted organic photovoltaics. *Chem. Sci.* **2025**, *16*, 17391-17407.
[24] G. Scalmani, M. J. Frisch, “Continuous surface charge polarizable continuum models of solvation. I. General formalism” *J. Chem. Phys.* **2021**, *132*, 114110.
[25] T. Jacopo, M. Benedetta, C. Roberto, “Quantum Mechanical Continuum Solvation Models” *Chem. Rev.* **2005**, *105*, 2999-3094.
[26] D. A. McQuarrie, J. D. Simon, *Molecular Thermodynamics*, *University Science Books*, **1999**, p. 159.
[27] G. R. Hutchison, M. A. Ratner, T. J. Marks, “Hopping Transport in Conductive Heterocyclic Oligomers: Reorganization Energies and Substituent Effects” *J. Am. Chem. Soc.* **2005**, *127*, 2339−2350.
[28] A. Thomas, R. K. Chitumalla, A. L. Puyad, K. V. Mohan, J. Jang, “Computational Studies of Hole/Electron Transport in Positional Isomers of Linear Oligo-Thienoacenes: Evaluation of Internal Reorganization Energies Using Density Functional Theory” *Comput. Theor. Chem.* **2016**, *10*, 59−67.
[29] L. Benatto, K. Marlus, “Effects of Fluorination on Exciton Binding Energy and Charge Transport of π‑Conjugated Donor Polymers and the ITIC Molecular Acceptor: A Theoretical Study” *J. Phys. Chem. C*. **2019**, *123*, 6395−6406.
[30] R. B. Ribeiro, M. T. D. N. Varella, “Excited state properties of an A–D–A nonfullerene electron acceptor: a LC-TD-DFTB study” *Phys. Chem. Chem. Phys.* **2024**, *26*, 12993.
[31] S. F. Nelsen, F. Blomgren, “Estimation of Electron Transfer Parameters from AM1 Calculations” *J. Org. Chem.* **2001**, *66*, 6551–6559.
[32] L. R. Franco, R. B. Ribeiro, H. M. Cezar, “Clusttraj: A Solvent-Informed Clustering Tool for Molecular Modeling” *J. Chem. Theory and Comput.* **2025** 21,6759-6768.
[33] G. T. Velde, F. M. Bickelhaupt, E. J. Baerends, C. F. Guerra, S. J. A. Gisbergen, J. G. Snijders, T. Ziegler, “Chemistry with ADF” *J. Comput. Chem*. **2001**, *22*, 931-967.
[34] J. Perdew, B. P. Kieron, E. Matthias, "Generalized gradient approximation made simple” *Physical review letters* **1996**, *77*, 3865.
[35] F. Jensen, “Polarization consistent basis sets: Principles” *J. Chem. Phys.* **2001**, *115*, 9113-9125.
[36] W. Zhu, A. P. Spencer, S. Mukherjee, J. M. Alzola, V. K. Sangwan, S. H. Amsterdam, S. M. Swick, L. O. Jones, M. C. Heiber, A. A. Herzing, G. Li, C. L. Stern, D. M. Delongchamp, K. L. Kohlstedt, M. C. Hersam, G. C. Schatz, M. R. Wasielewski, L. X. Chen, A. Fecchetti, T. J. Marks, “Crystallography, Morphology, Electronic Structure, and Transport in Non-Fullerene/Non-Indacenodithienothiophene Polymer:Y6 Solar Cells” *J. Am. Chem. Soc.* **2020**, *142*, 14532–14547.
[37] L. Benatto, M. Koehler, “Effects of Fluorination on Exciton Binding Energy and Charge Transport of π‐Conjugated Donor Polymers and the ITIC Molecular Acceptor: A Theoretical Study” *J. Phys. Chem. C* **2019**, *11*, 6395-6406.
[38] N. Michaud-Agrawal, E. J. Denning, T. B. Woolf, O. Beckstein, “MD Analysis: A toolkit for the analysis of molecular dynamics simulations” *J. Comput. Chem.* **2011**, *32*, 2319–2327.
[39] A. A. Hagberg, D. A. Schult, P. J. Swart, Exploring network structure, dynamics, and function using Network X. *Proceedings of the 7th Python in Science Conference*. **2008**, p. 11–15.
[40] C. R. Harris, K. J. Millman, S. J. van der Walt, R. Gommers, P. Virtanen, D. Cournapeau, E. Wieser, J. Taylor, S. Berg, N. J. Smith, R. Kern, M. Picus, S. Hoyer, M. H. van Kerkwijk, M. Brett, A. Haldane, J. F. D. Rio, M. Wiebe, P. Peterson, P. G. Marchant, K. Sheppard, T. Reddy, W. Weckesser, H. Abbasi, C. Gohlke, T. E. Oliphant, ”Array programming with NumPy” *Nature*. **2020**, *585*, 357–362.
[41] [41] W. McKinney, “Data structures for statistical computing in Python” *Proceedings of the 9th Python in Sci. Conference* **2010**, 56–61.
[42] [42] J. Hunter, D. Matplotlib, “A 2D graphics environment” *Comput. Sci. Eng.* **2007**, *9*, 90–95.
[43] G. Zhang, X. Chen, J. Xiao, C. Y. C. Philip, M. Ren, G. Kupgan, X. Jiao, C. C. S. Chan, X. Du, R. Xia, Z. Chen, J. Yuan, Y. Zhang, S. Zhang, Y. Liu, Y. Zou, H. Yan, K. S. Wong, V. Coropceanu, N. Li, C. J. Brabec, J.-L. Bredas, Y.-L. Yip, Y. Cao, “Delocalization of exciton and electron wavefunction in non-fullerene acceptor molecules enables efficient organic solar cells” *Nat. Commun.* **2020**, *11*, 3943.
[44] M. Andrea, K. Kordos, E. Lidorikis, D. Papageorgiou, “Fluorination and chlorination effects on the charge transport properties of the IDIC non-fullerene acceptor: an ab-initio investigation” *EPJ Photovoltaics* **2022**, *13*, 1-10.
